# Supplementary material for: Blood circulation of soft nanomaterials is governed by dynamic remodeling of protein opsonins at nano-biointerface
Source: Nat Commun. 2020 Jun 16;11:3048. doi: 10.1038/s41467-020-16772-x (PMC7298025; doi:10.1038/s41467-020-16772-x)
Supplement: Supplementary file 1 — Supplementary Information [file 41467_2020_16772_MOESM1_ESM.pdf]

Blood circulation of soft nanomaterials is governed by dynamic remodeling of protein opsonins at nanobiointerface

Srinivas Abbina[1,2]†, Lily E. Takeuchi[1,2]†, Parambath Anilkumar[1,2]†, Kai Yu[1,2], Jason C. Rogalski[3], Rajesh A. Shenoi[4], Iren Constantinescu[1,2], Jayachandran N. Kizhakkedathu[1,2,5]*

Correspondence to: jay@pathology.ubc.ca

This PDF file includes:

Supplementary Figures. 1 to 13
Supplementary Tables. 1 to 11

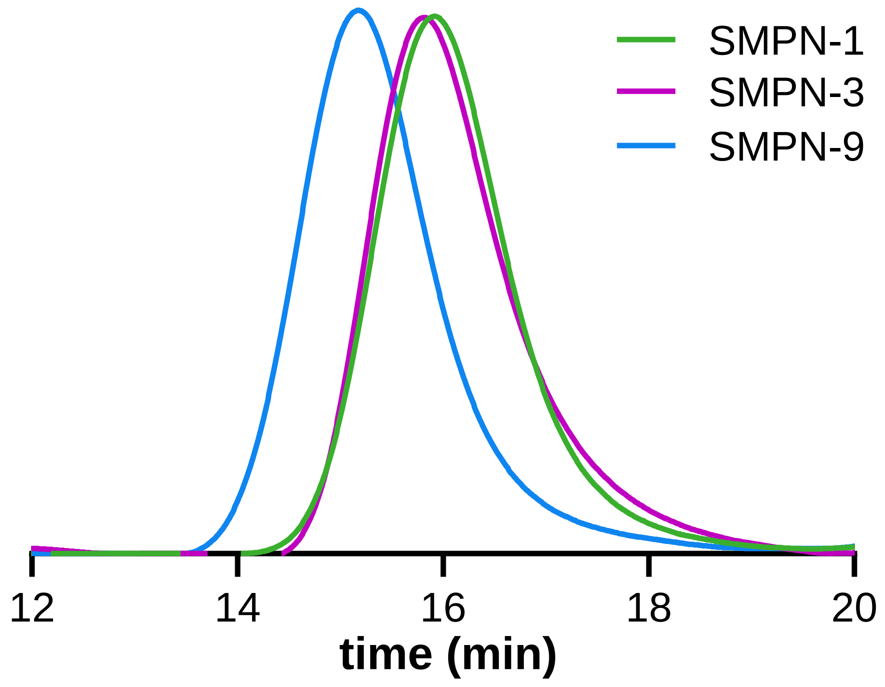

Supplementary Figure 1. Determination of absolute molecular weight of SMPNs. Molecular weight (Mw) of the SMPNs was determined by gel permeation chromatography (GPC) coupled with multi angle laser light scattering (MALS) and refractive index detectors. The absolute molecular weights (weight average molecular weight (Mw)) of the SMPN-1, SMPN-3, and SMPN-9 determined from the analysis were 1.3, 2.9, and 9.3 million Daltons respectively.

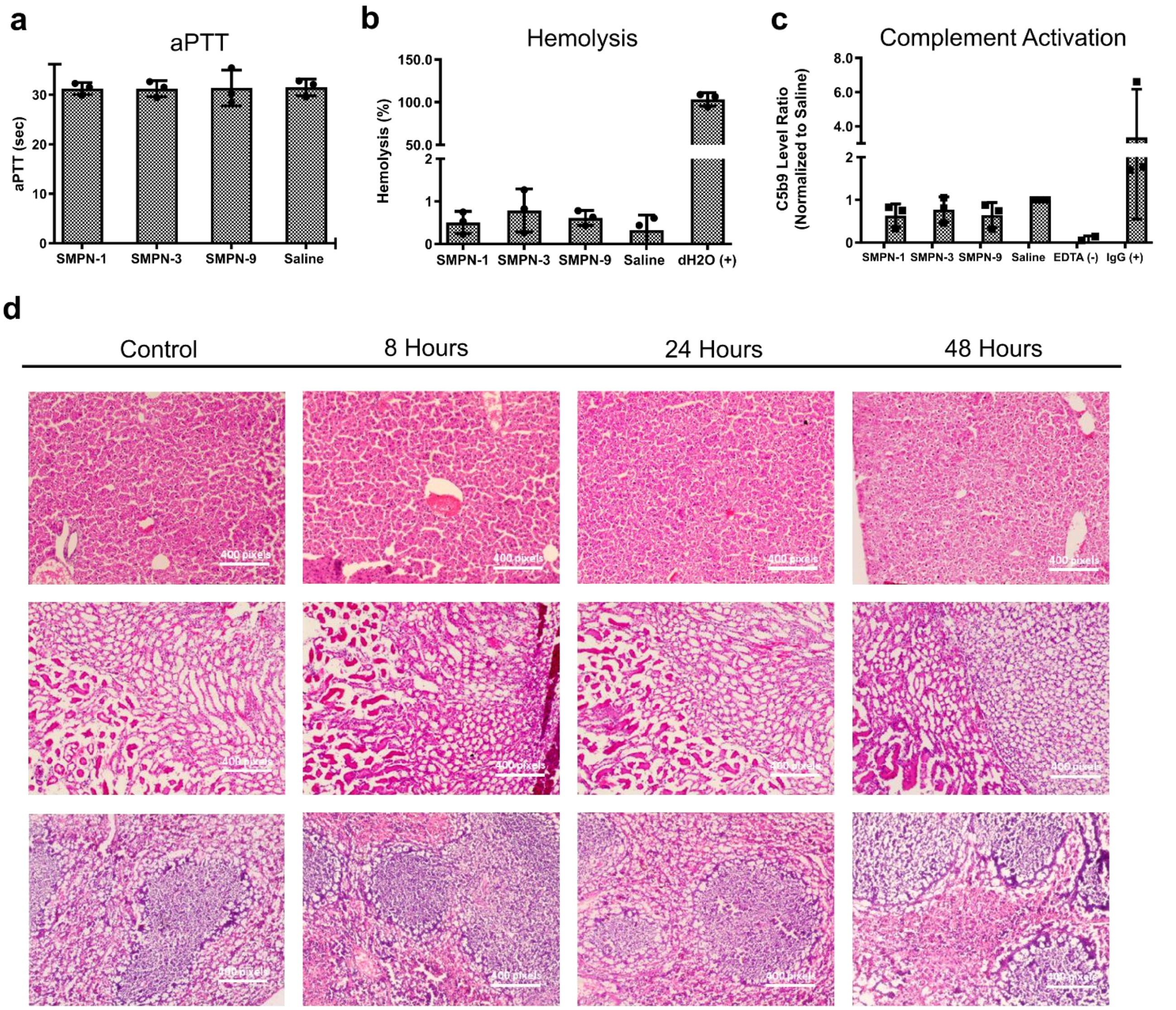

Supplementary Figure 2. Biocompatibility of SMPNs. Two-sided t-tests were performed for all blood biocompatibility tests to compare with saline controls, no significant differences were generated, thus SMPNs showed no adverse effects on **(a)** blood coagulation activated partial thromboplastin time, **(b)** complement activation (mean ± s.d., n = 3 donor experimental replicates). In complement activation assays, EDTA is used as a negative control and IgG is used as a positive control. **(c)** Further, samples show no significant differences red blood cell lysis (mean ± s.d., n = 3 donor experimental replicates) compared to saline controls. **(d)** Histological sections of tissues after intravenous injection of SMPN-1 at 500 mg/kg is followed over 2 days. The tissue sections were stained using H&E stain. SMPN-1 did not demonstrate overt signs of toxicity when assessed histologically of liver, kidney, or spleen after intravenous injection of SMPN-1. The organs were collected and fixed using paraformaldehyde. A representative set of images were shown here from n = 4 mice (5 images were per mouse).

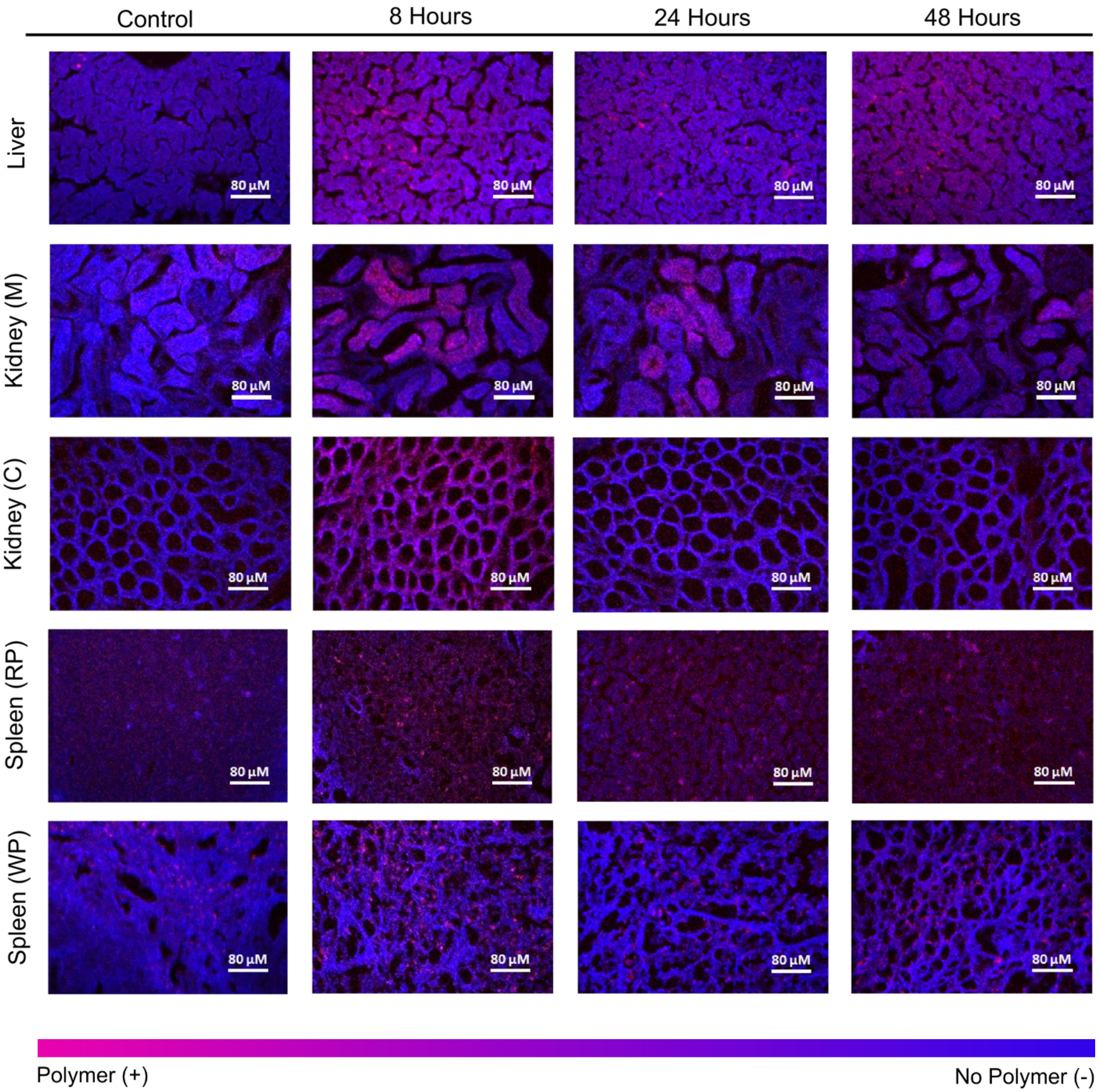

Supplementary Figure 3. Qualitative comparison of organ accumulation of SMPN-1 at 8, 24, and 48 h. Spinning disk confocal microscopy analysis was used for the organ accumulation analysis of fluorescently labeled SMPN-1 at 8 and 48 h. The organs were collected from mice at 8 & 48 h after intravenous administration SMPN-1, fixed, and frozen organs were sliced to 100 micrometer sections before being analyzed by spinning disk confocal microscopy. Representative tissue sections are shown. The data showed that the accumulation of SMPN-1 was increased in the liver, kidney (cortex), and spleen (white pulp) with time. See quantitative analysis in Fig. 2C. 40 images collected for each group (10 images per mice; n = 4 mice). A representative set of images were shown here.

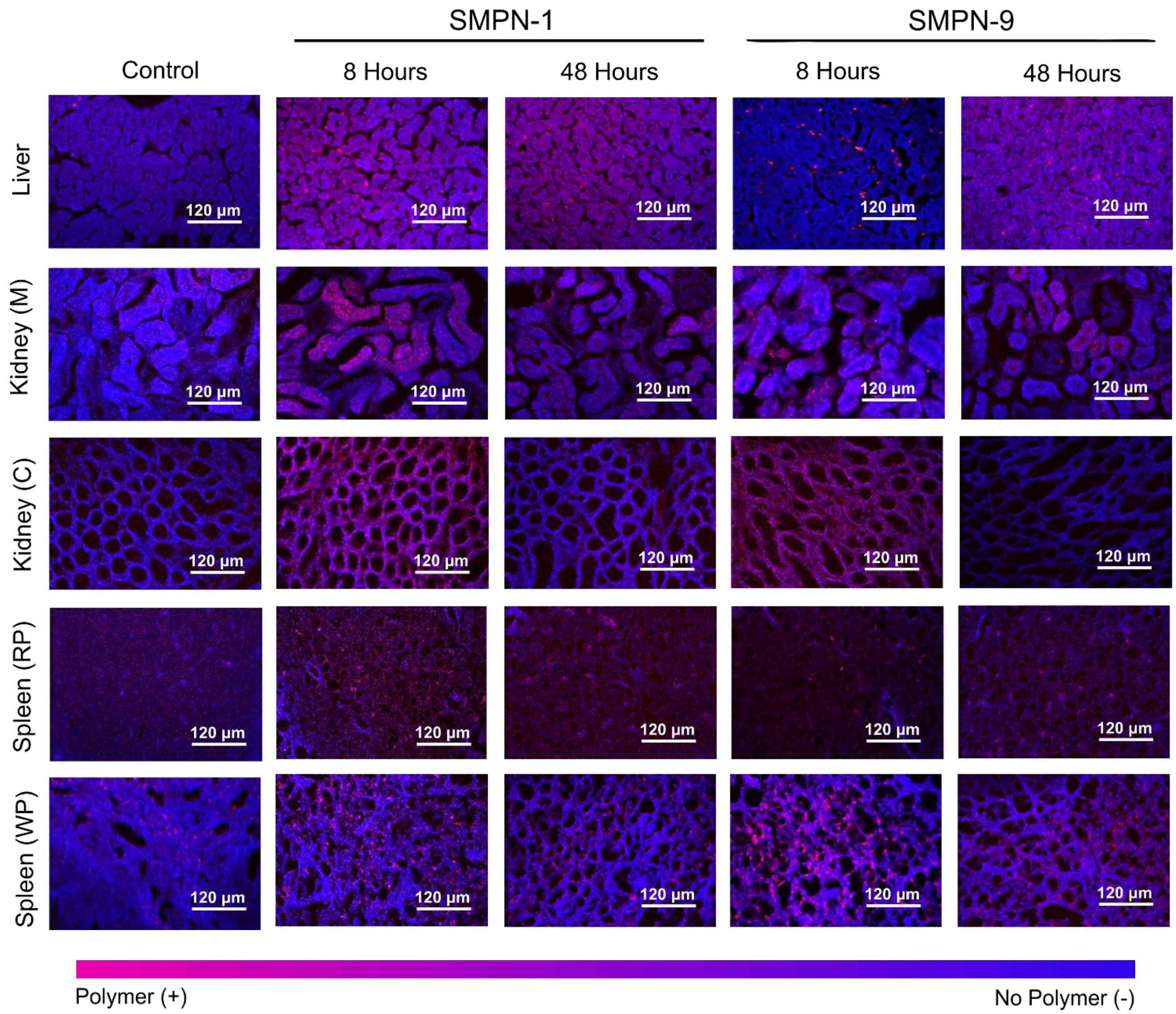

Supplementary Figure 4. Qualitative comparison of organ accumulation of SMPN-1 and -9. Spinning disk confocal microscopy analysis was used for the organ accumulation analysis of fluorescently labeled SMPN-1, and -9 at 8 and 48 h. The organs were collected from mice at 8 & 48 h after intravenous administration, fixed and frozen organs were sliced to 100 micrometer sections before being analyzed by confocal microscopy. Representative tissue sections are shown. The data demonstrate that SMPN-1 and -9 are localized differentially in the liver, kidney, and spleen (see also Fig. 2 in the main text for quantitative comparison from multiple tissue sections). 40 images collected for each group (10 images per mice; n = 4 mice). A representative set of images were shown here.

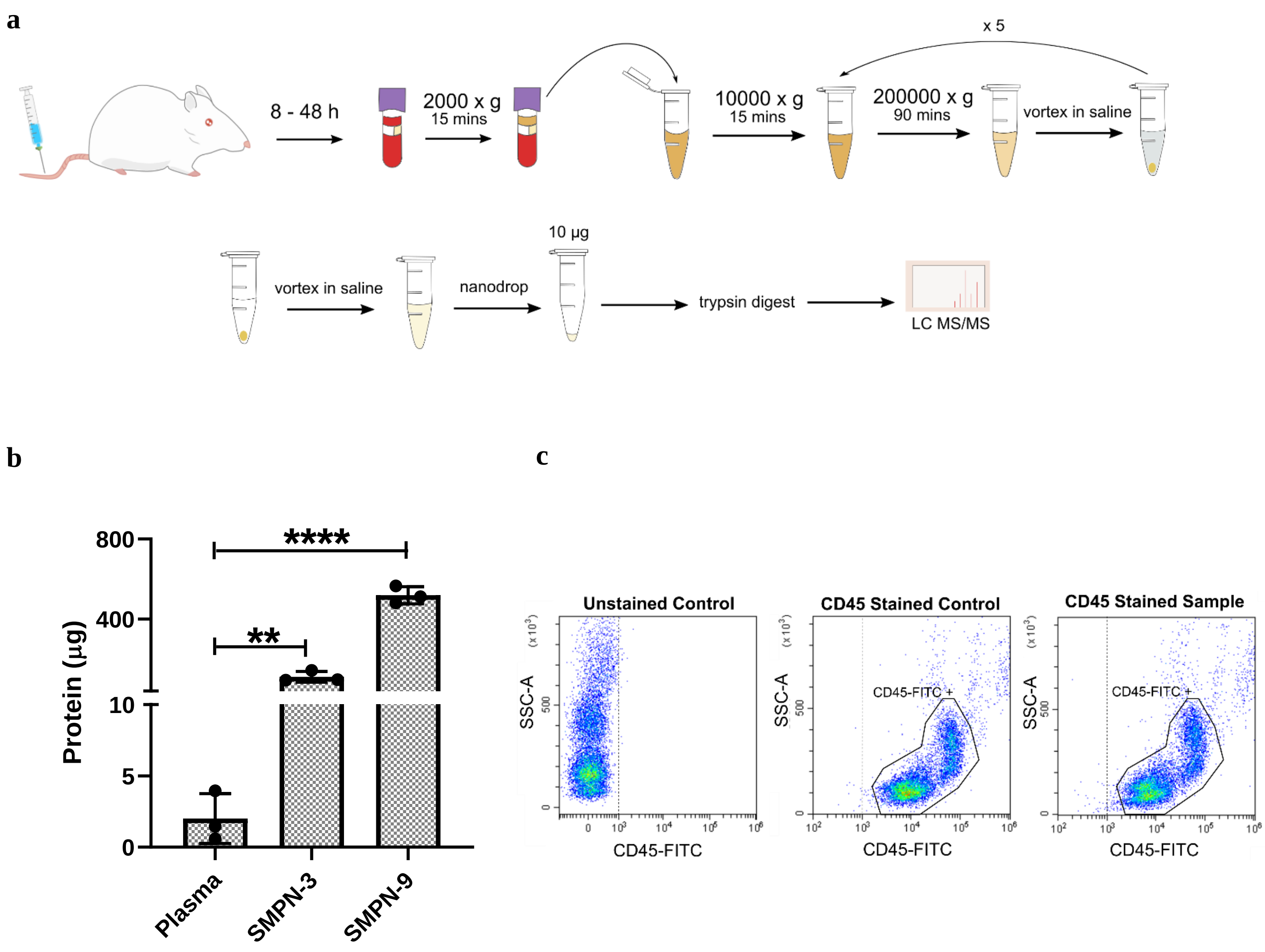

Supplementary Figure 5. The workflow of SMPN isolation. (a) SMPN isolation was performed by collecting whole blood from mice in EDTA tubes. Next, tubes were centrifuged at 2000 g for 15 minutes for plasma collection. Plasma was collected into a new tube and centrifuged at 10,000 g for 15 minutes to remove any additional microparticles and debris. Next, plasma was ultracentrifuge at 200,000 g for 90 minutes. The supernatant was removed and the polymer pellet was resuspended (vortexed) in saline until homogenously distributed. This washing step was repeated 5 times before collecting the final pellet in 200 µL of saline. Protein collections were measured by NanoDrop and 10 µg aliquots were collected and subjected to trypsin digest and C18 column purification before mass spectrometry analysis. **(b)** The SMPNs (500 mg/kg) were incubated in human plasma for 1 h and were isolated by our sequential

centrifuging protocol. Pure plasma was also subjected to the same treatment. The protein content (mean ± s.d.) of the isolated SMPNs from three independent experiments with replicates was determined by NanoDrop™ UV-Vis Spectrophotometer and compared it with pure plasma. One-sided t-tests were performed to determine the abundance of the proteins on SMPNs was quite significant than pure plasma alone (**p = 0.003746, plasms Vs SMPN-3; ****p = 0.000039, plasms Vs SMPN-9). **(c)** Flow cytometry plots indicating successful identification of CD45+ leukocyte populations (10000 cells per mouse, n = 4 mice) with >90% purity is depicted.

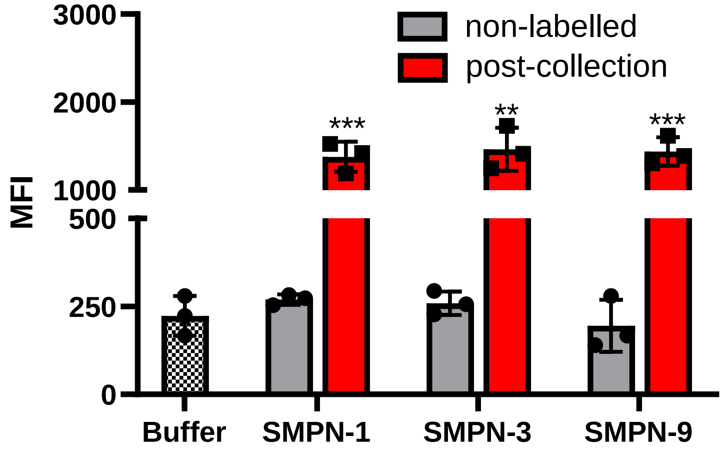

Supplementary Figure 6. Confirmation of SMPNs isolation. Isolation of SMPNs was confirmed using fluorescence-based methods. Protein corona-SMPN isolates after the 5 washes collected from mice injected with Hilyte Fluor 647-labelled SMPNs were collected and fluorescence was measured and compared to native SMPN (with no label). Fluorescence intensity (MFI) at 670 nm (mean ± s.d., N = 3 mice) are plotted. One-sided t-test performed to compare non-labelled (native) and post-collection SMPNs demonstrate MFI from all SMPN groups (SMPN-1 ***p = 0.004; SMPN-3 **p = 0.001; SMPN-9 ***p = 0.0003) were significantly higher than that of native SMPN and buffer control suggesting that SMPNs were successfully isolated by the sequential centrifugation and washing protocol.

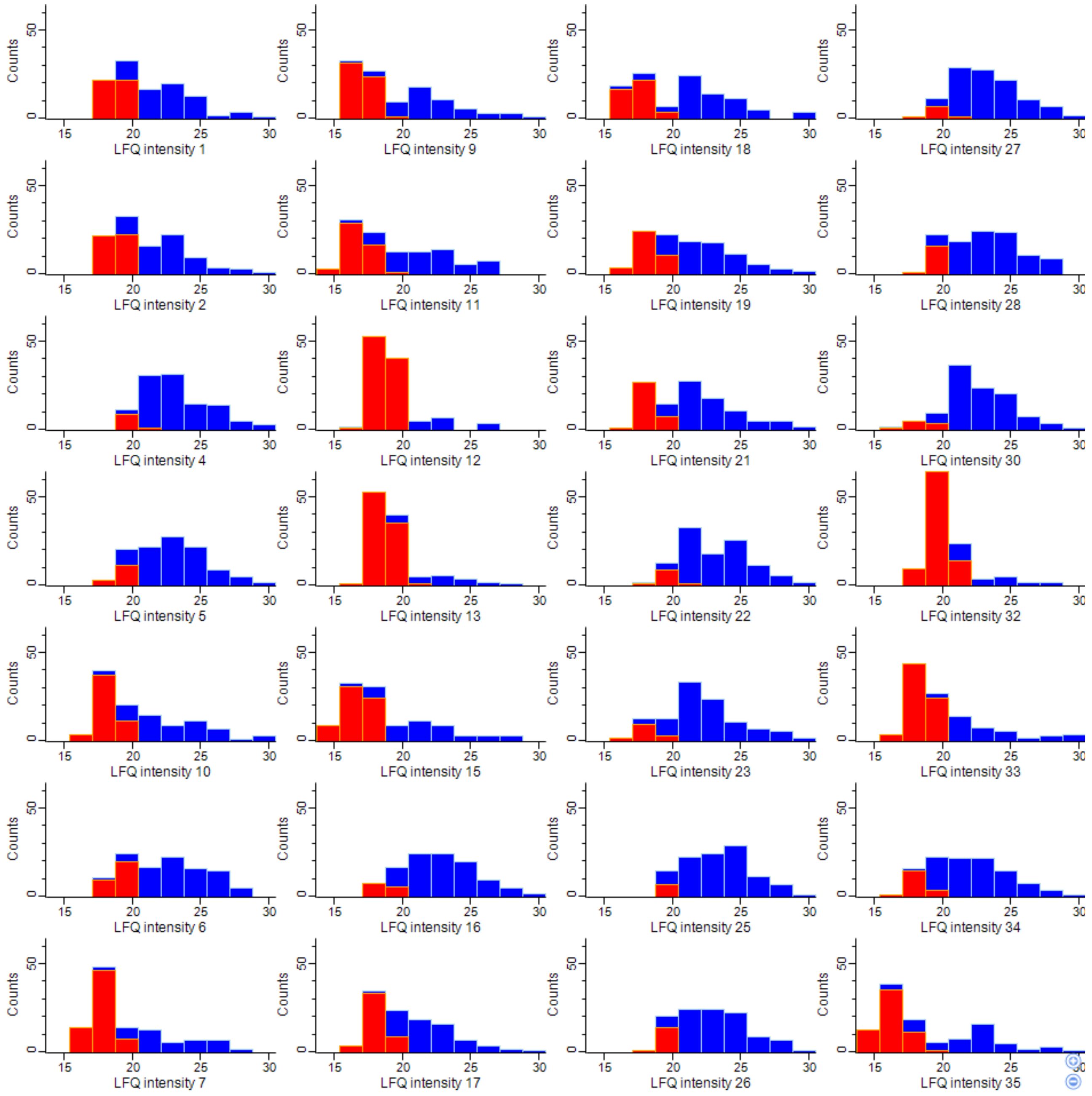

Supplementary Figure 7. Histograms depicting the number of proteins (y axis) and the log2-transformed LFQ intensities for each of the proteins. Identified/imputed for each of the mice (indicated by number given X-axis labels). Mean LFQ intensities are derived from 3 technical replicates. Red bars indicate imputed data while blue bars indicate non-imputed data.

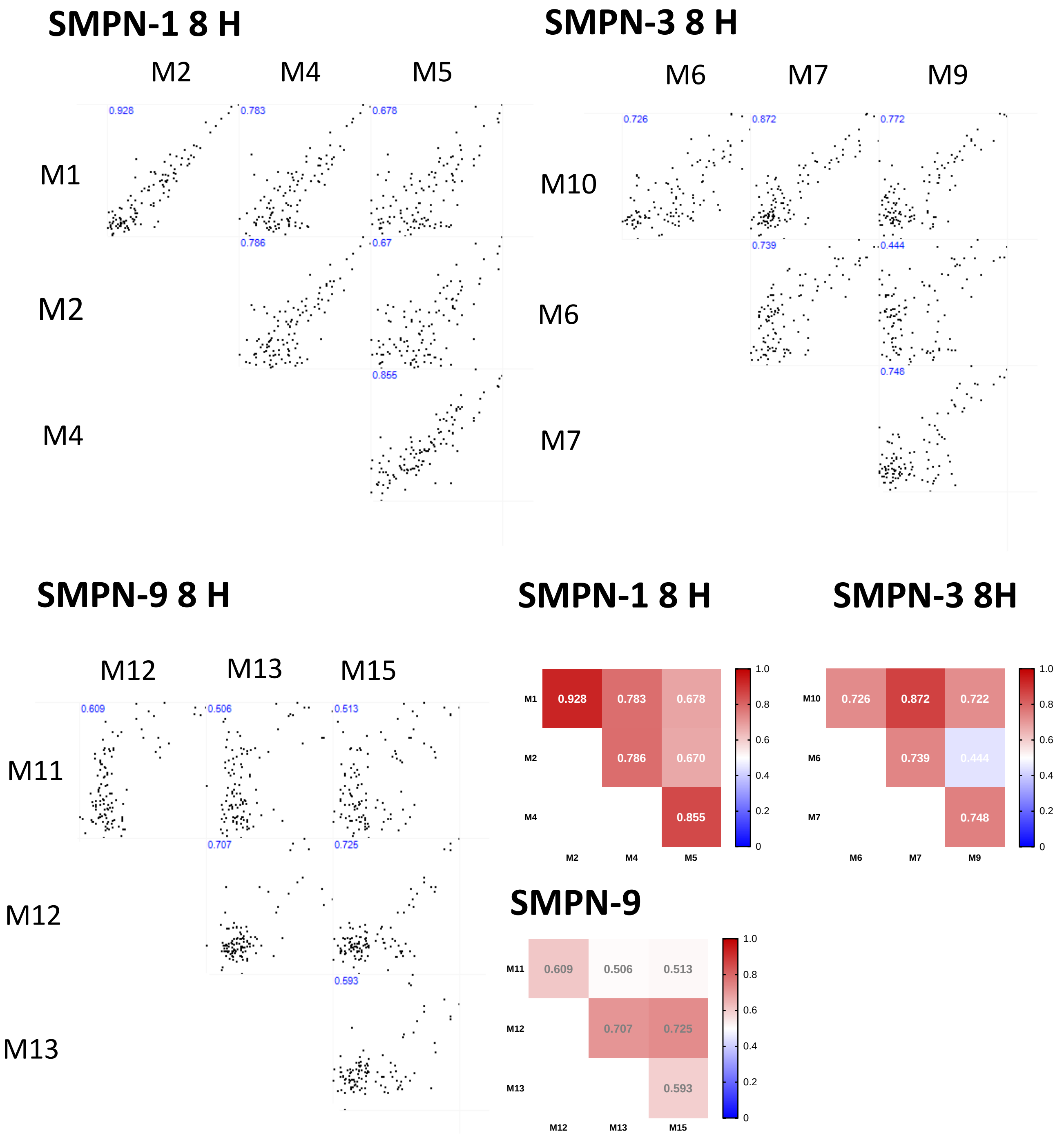

Supplementary Figure 8. Binary scatterplots for SMPNs at 8 h. Scatterplots demonstrating each of the mean (N = 3) LFQ intensities for each mouse (M#) compared against mice in the same group. Pearson correlation coefficients are reported in histograms in the bottom right-hand corner.

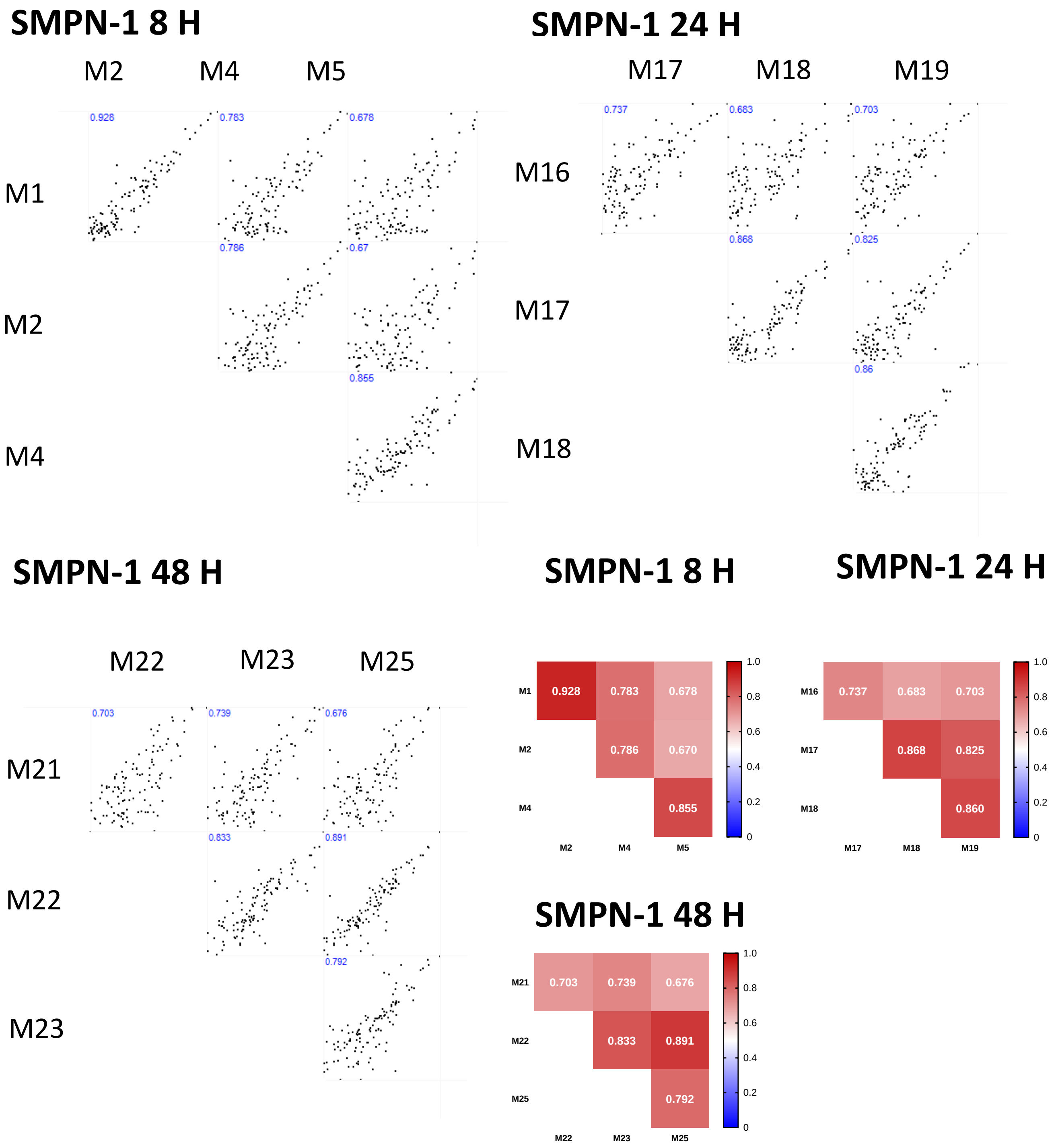

Supplementary Figure 9. Binary scatterplots for SMPN-1 at various time points. Scatterplots demonstrating each of the mean (N = 3) LFQ intensities for each mouse (M#) compared against mice in the same group. Pearson correlation coefficients are reported histograms in the bottom right hand corner.

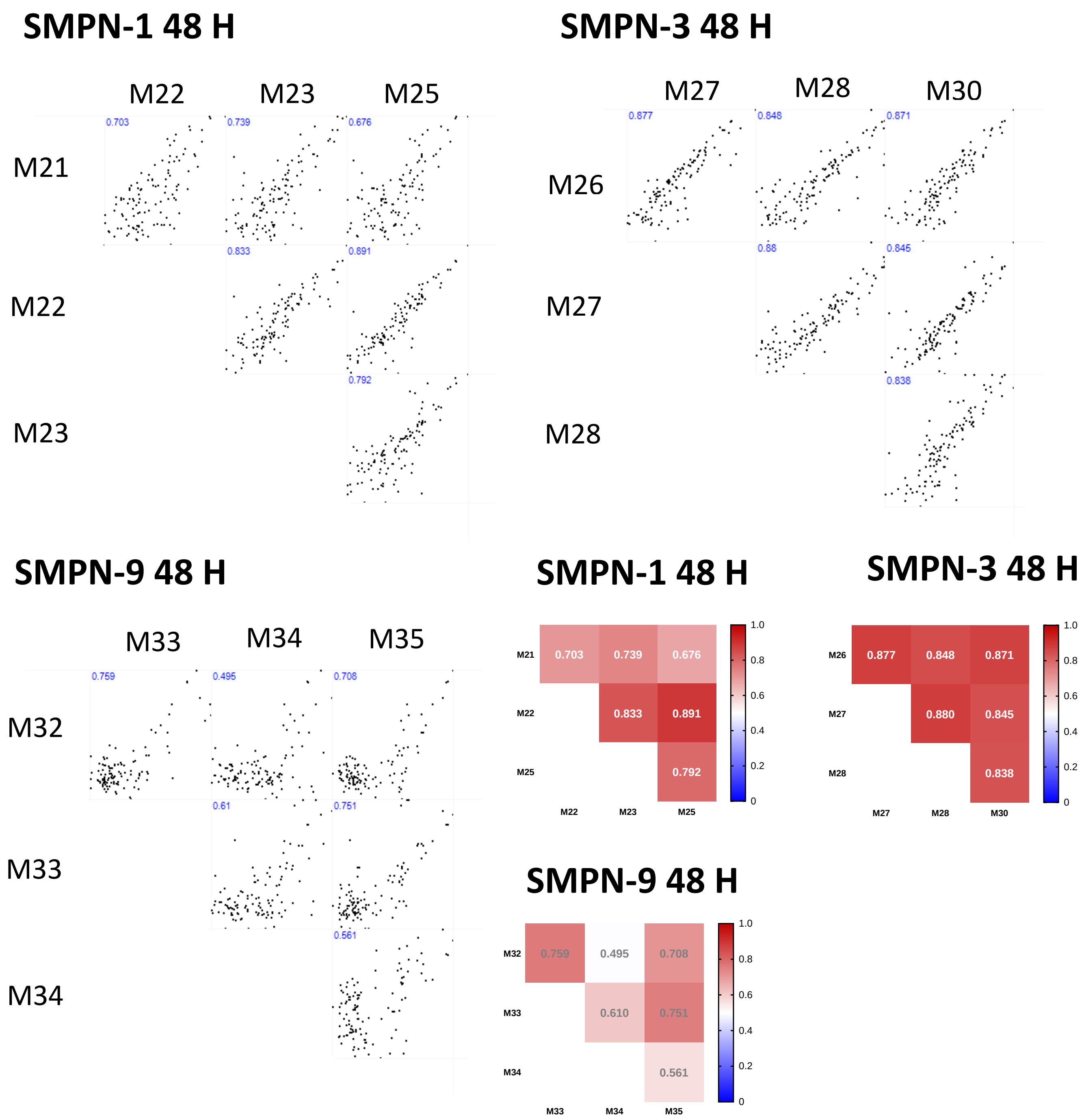

Supplementary Figure 10. Binary scatterplots for SMPNs at 48 h. Scatterplots demonstrating each of the mean (N =3) LFQ intensities for each mouse (M#) compared against mice in the same group. Pearson correlation coefficients are reported in histograms in the bottom right hand corner.

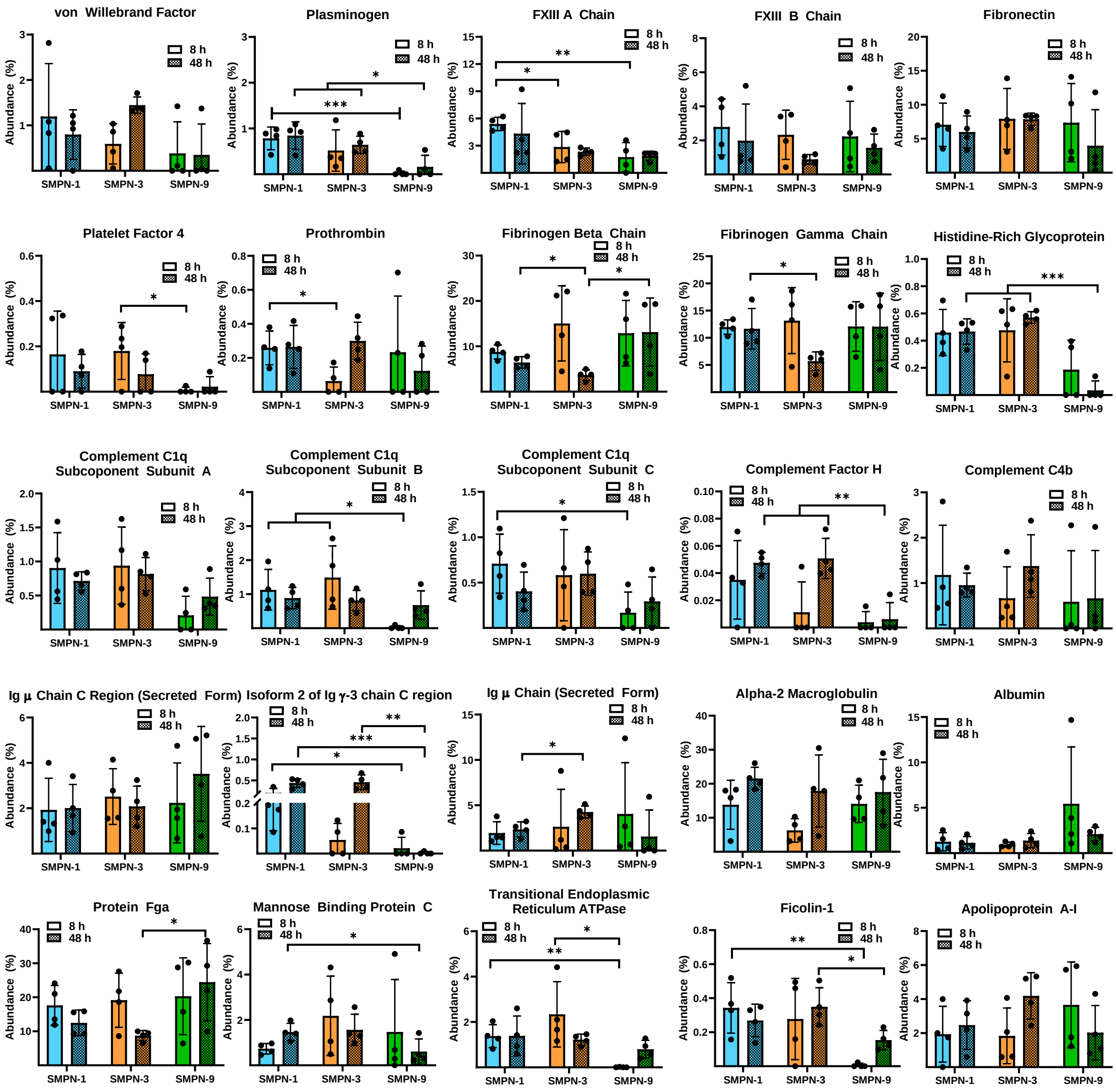

Supplementary Figure 11. Bar graphs of the top 25 must abundant commonly shared proteins among SMPN-1, -3, and -9 at 8 and 48 h. The presented proteomics workflow is a robust method to quantify differences in corona proteins. Two-sided t-tests' were conducted between various molecular weight SMPN groups and significant values are noted (*$p < 0.05$, **$p < 0.01$, ***$p < 0.001$). Mean protein abundance values ± s.d. from N = 4 mice are shown.

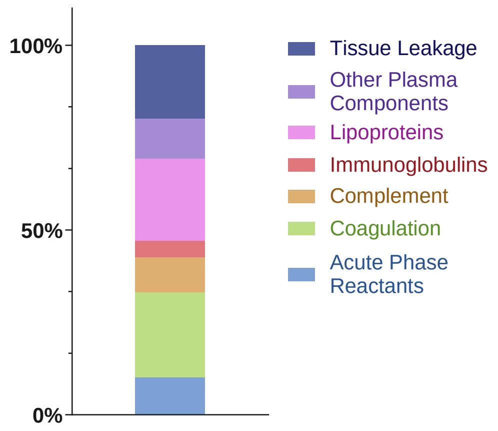

Supplementary Figure 12. Identification of proteins in normal mouse plasma. Proteomic analysis conducted on pooled mouse plasma containing 0.8% sodium citrate demonstrated higher content lipoproteins, coagulation proteins, and tissue leakage proteins followed by acute phase reactants, complement proteins, and immunoglobulins. The plasma was not exposed to SMPNs.

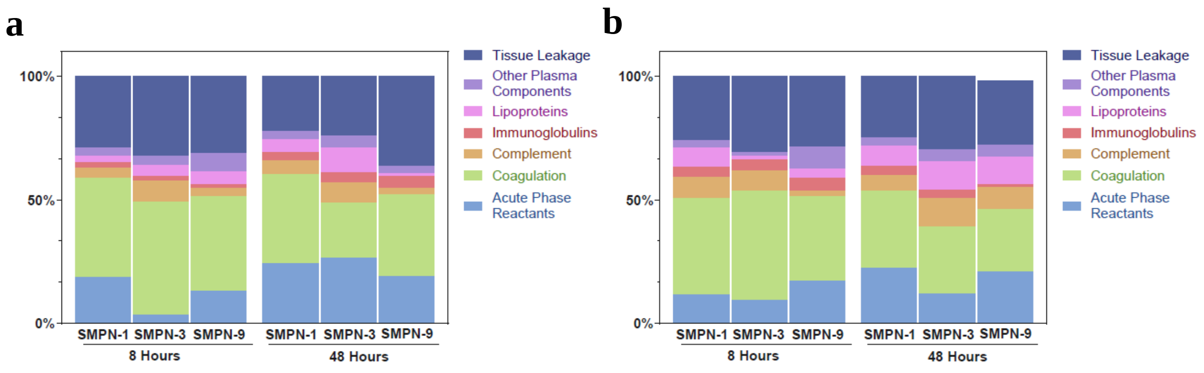

Supplementary Figure 13. Analysis of protein corona on different SMPNs isolated from male and female mice. The figure is depicting average percent abundance of corona proteins classified by functional group in (N = 2) female mice (**a**) and (N = 2) male mice (**b**) at two time points. Please also see figure 4 in the main text for more detailed analysis.

Sample ID	Amount of glycidol (mL)	M_w(million Da)	DOB	Yield (%)
SMPN-1	8.0	1.3	0.57	74
SMPN-3	24	2.9	0.54	85
SMPN-9	51	9.3	0.53	74

Supplementary Table 1. Amount of glycidol used to generate SMPNs with different molecular weights, size, yields, and degree of branching (DOB) are given.

sample ID	α (h^{-1})	β (h^{-1})	$t_{1/2\beta}$ (h)	P_o (mg/mL)	V_c (mL)	k_{12} (h^{-1})	k_{21} (h^{-1})	k_2(h^{-1})	$AUC_{0\rightarrow\infty}$ (mg/mL/h)
**SMPN-1**	0.217±0.007	0.011±0.001	65.54±5.73	0.417±0.030	0.048±0.003	0.060±0.079	0.152±0.008	0.015±0.006	27.64
**SMPN-3**	0.734±0.274	0.012±0.001	57.80±1.39	0.436±0.046	0.046±0.005	0.249±0.287	0.479±0.085	0.018±0.008	23.68
**SMPN-9**	0.204±0.001	0.031±0.004	22.14±2.60	0.220±0.015	0.091±0.006	0.018±0.006	0.182±0.003	0.035±0.004	6.28

Supplementary Table 2. Pharmacokinetic parameters of SMPNs. All pharmacokinetic parameters were calculated based off a two-compartment model. SMPN-9 gave lower half-life, lower P_o, higher k_2 and lower AUC compared to SMPN-1 or SMPN-3, all indicating faster elimination from blood circulation. All the data is reported as mean ± s.d.

$t_{1/2\beta}$ Elimination half life;
P_o Initial polymer concentration in plasma
V_c Plasma volume estimate
k_{12} Rate constant for blood to tissue
k_{21} Rate constant for tissue to blood
k_2 Rate constant for elimination
AUC Area under the curve

Sample ID	Relative fluorescence units (RFU)
SMPN-1	41597 ± 482
SMPN-3	33533 ± 1448
SMPN-9	44342 ± 961

Supplementary Table 3. Quantification of fluorescence intensity of SMPNs. Fluorescence quantification was performed on SpectraMax microplate reader (Molecular Devices) using an excitation wavelength of 649 nm. Data is presented as a mean ± s.d. of six measurements.

Sample	Time in circulation (h)	Protein content (mg/mL)
SMPN-1	8	0.061 ± 0.045
SMPN-1	24	0.175 ± 0.133
SMPN-1	48	0.032 ± 0.026
SMPN-3	8	0.279 ± 0.263
SMPN-3	48	0.030 ± 0.020
SMPN-9	8	0.403 ± 0.365
SMPN-9	48	0.266 ± 0.191

Supplementary Table 4. Adsorbed protein content on SMPNs measured after isolation from mice. SMPNs were isolated from mouse blood at different time points (SMPN-1: 8, 24, and 48 h and SMPN-3 & 9: 8 and 48 h) after intravenous administration. Duplicate measurements were done in saline for each sample and the values were reported as mean protein content ± s.d. measured across three different nanodrop instruments.

Supplementary Table 5. Top 25 proteins on SMPN-1 at various time points. Four independent biological replicates at different time points are shown.

	SMPN-1 (8 h)				SMPN-1 (24 h)				SMPN-1 (48 h)			
1	Protein Fga (21.0%)	Protein Fga (24.1%)	α-2-macroglobulin (18.2%)	Protein Fga (13.5%)	Fibronectin (16.2%)	α-2-macroglobulin (31.7%)	Protein Fga (23.4%)	α-2-macroglobulin (33.2%)	α-2-macroglobulin (18.4%)	α-2-macroglobulin (26.0%)	α-2-macroglobulin (21.7%)	α-2-macroglobulin (20.2%)
2	α-2-macroglobulin (18.0%)	α-2-macroglobulin (15.9%)	Protein Fga (11.9%)	Fibrinogen γ chain (11.7%)	α-2-macroglobulin (16.1%)	Protein Fga (15.9%)	α-2-macroglobulin (20.4%)	Protein Fga (16.5%)	Fibrinogen γ chain (17.0%)	Protein Fga (9.2%)	Protein Fga (15.9%)	Fibrinogen γ chain (9.4%)
3	Fibrinogen γ chain (12.5%)	Fibrinogen γ chain (13.4%)	Fibrinogen γ chain (10.3%)	Fibronectin (11.3%)	Protein Fga (8.0%)	Fibrinogen γ chain (10.6%)	Fibrinogen β chain (17.1%)	Fibrinogen γ chain (11.8%)	Protein Fga (15.6%)	Fibrinogen γ chain (8.9%)	Fibrinogen γ chain (11.3%)	Protein Fga (9.2%)
4	Fibrinogen β chain (9.1%)	Fibrinogen β chain (8.6%)	Fibrinogen β chain (6.8%)	Fibrinogen β chain (10.5%)	Fibrinogen γ chain (7.2%)	Fibrinogen β chain (8.1%)	Fibrinogen γ chain (16.6%)	Fibrinogen β chain (8.8%)	FXIII A chain (9.2%)	Fibronectin (5.2%)	Fibronectin (8.9%)	Fibronectin (6.5%)
5	Fibronectin (7.0%)	FXIII A chain (6.2%)	Fibronectin (6.5%)	FXIII A chain (4.9%)	Gelsolin (5.5%)	FXIII A chain (5.0%)	FXIII B chain (3.1%)	Fibronectin (4.0%)	Fibrinogen β chain (7.3%)	Fibrinogen β chain (5.2%)	Fibrinogen β chain (7.8%)	Fibrinogen β chain (5.5%)
6	FXIII A chain (5.8%)	FXIII B chain (4.4%)	FXIII A chain (4.6%)	Apolipoprotein A-I (4.0%)	Fibrinogen β chain (5.2%)	FXIII B chain (3.3%)	Ig µ chain C region secreted form (2.7%)	FXIII A chain (3.1%)	FXIII B chain (5.2%)	Apolipoprotein A-I (3.9%)	Apolipoprotein A-IV (3.0%)	FXIII A chain (3.6%)
7	FXIII B chain (3.9%)	Fibronectin (3.5%)	Ig µ chain C region secreted form (4.0%)	Gelsolin (3.8%)	FXIII A chain (4.2%)	Fibronectin (3.0%)	FXIII A chain (2.7%)	Complement C3 (2.9%)	Fibronectin (3.3%)	Gelsolin (3.2%)	Gelsolin (2.6%)	Ig µ chain C region secreted form (3.4%)
8	Complement C3 (1.9%)	Serum albumin (2.5%)	Apolipoprotein A-I (3.2%)	α-2-macroglobulin (3.1%)	Complement C3 (3.1%)	Complement C3 (2.7%)	Fibronectin (2.6%)	Ig µ chain C region secreted form (2.5%)	Transitional endoplasmic reticulum ATPase (2.6%)	Complement C3 (3.1%)	Apolipoprotein A-I (2.2%)	Apolipoprotein A-I (3.2%)
9	Apolipoprotein A-I (1.7%)	Apolipoprotein A-I (2.0%)	Transitional endoplasmic reticulum ATPase (2.0%)	von Willebrand factor (2.8%)	Apolipoprotein A-I (2.4%)	Mannose-binding protein C (1.9%)	Galactose-3-O-sulfotransferase 3 (1.6%)	FXIII B chain (2.4%)	Ig µ chain C region secreted form (2.0%)	FXIII A chain (2.2%)	FXIII A chain (2.2%)	Complement C3 (2.6%)
10	Serum albumin (1.6%)	Complement C3 (1.7%)	Complement C1q subcomponent subunit B (2.0%)	Complement C4-B (2.8%)	Mannose-binding protein C (2.1%)	Ig µ chain C region secreted form (1.7%)	Complement C3 (1.4%)	Apolipoprotein A-I (1.3%)	Mannose-binding protein C (1.4%)	Serum albumin (2.1%)	Mannose-binding protein C (2.0%)	Gelsolin (2.3%)
11	Ig µ chain C region secreted form (1.4%)	Gelsolin (1.5%)	Complement C3 (1.8%)	Apolipoprotein E (1.4%)	Apolipoprotein A-IV (1.8%)	α-2-macroglobulin-P (1.3%)	Mannose-binding protein C (0.6%)	CD5 antigen-like (1.2%)	Complement C3 (1.4%)	Ceruloplasmin (1.9%)	Complement C3 (1.9%)	Complement C4-B (1.3%)
12	α-2-macroglobulin-P (1.1%)	Transitional endoplasmic reticulum ATPase (1.3%)	FXIII B chain (1.8%)	Transitional endoplasmic reticulum ATPase (1.4%)	FXIII B chain (1.4%)	α-amylase 1 (1.3%)	Plasminogen (0.5%)	Complement C4-B (1.1%)	Gelsolin (1.3%)	Ig µ chain C region secreted form (1.7%)	Transitional endoplasmic reticulum ATPase (1.4%)	Apolipoprotein A-IV (1.2%)
13	Gelsolin (1.0%)	Ig µ chain C region secreted form (1.0%)	Complement C1q subcomponent subunit A (1.6%)	Ig µ chain C region secreted form (1.3%)	Complement C1q subcomponent subunit B (1.4%)	von Willebrand factor (1.1%)	Serum albumin (0.4%)	Mannose-binding protein C (0.8%)	Complement C1q subcomponent subunit B (1.2%)	Mannose-binding protein C (1.4%)	Ig µ chain C region secreted form (0.9%)	Murinoglobulin-1 (1.2%)
14	Mannose-binding protein C (1.0%)	Plasminogen (0.8%)	Gelsolin (1.4%)	Complement C3 (1.3%)	Complement C4-B (1.4%)	Apolipoprotein A-I (1.1%)	Apolipoprotein A-I (0.4%)	Histidine-rich glycoprotein (0.6%)	von Willebrand factor (1.2%)	Apolipoprotein C-I (1.1%)	Isoform 7 of Nuclear factor 1 C-type (0.9%)	Serum albumin (1.2%)
15	Plasminogen (0.9%)	Complement C1q subcomponent subunit B (0.8%)	Murinoglobulin-1 (1.3%)	Complement C1q subcomponent subunit B (1.1%)	Transitional endoplasmic reticulum ATPase (1.3%)	Plasminogen (0.9%)	Complement C4-B (0.4%)	Plasminogen (0.6%)	Plasminogen (1.0%)	Plasminogen (1.1%)	Serum albumin (0.8%)	FXIII B chain (1.1%)

Supplementary Table 5. Top 25 proteins on SMPN-1 at various time points. Four independent biological replicates at different time points are shown (cont.).												
	SMPN-1 (8 h)				SMPN-1 (24 h)				SMPN-1 (48 h)			
**16**	von Willebrand factor (0.8%)	Mannose-binding protein C (0.6%)	Complement C1q subcomponent subunit C (1.1%)	Inter-α-trypsin inhibitor heavy chain H1 (1.1%)	Apolipoprotein E (1.2%)	Complement C1q subcomponent subunit B (0.7%)	von Willebrand factor (0.4%)	Complement C1q subcomponent subunit B (0.6%)	α-2-macroglobulin-P (0.9%)	Apolipoprotein E (1.0%)	Apolipoprotein C-III (0.8%)	Complement C1q subcomponent subunit B (1.1%)
**17**	Transitional endoplasmic reticulum ATPase (0.8%)	Serine protease inhibitor A3K (0.6%)	von Willebrand factor (1.1%)	FXIII B chain (1.0%)	Inter-α-trypsin inhibitor heavy chain H1 (1.2%)	Serum albumin (0.7%)	Ig kappa chain V-II region 26-10 (0.3%)	Clusterin (0.5%)	Complement C4-B (0.9%)	Inter-α-trypsin inhibitor heavy chain H2 (0.9%)	Complement C4-B (0.8%)	von Willebrand factor (1.1%)
**18**	Galactose-3-O-sulfotransferase 3 (0.8%)	Complement C1q subcomponent subunit C (0.6%)	Plasminogen (1.0%)	Complement C1q subcomponent subunit A (1.0%)	Inter-α-trypsin inhibitor heavy chain H2 (1.2%)	Ceruloplasmin (0.7%)	α-2-macroglobulin-P (0.3%)	Serum albumin (0.5%)	Complement C1q subcomponent subunit A (0.8%)	von Willebrand factor (0.9%)	FXIII B chain (0.8%)	Mannose-binding protein C (1.0%)
**19**	Histidine-rich glycoprotein (0.7%)	Complement C1q subcomponent subunit A (0.6%)	Apolipoprotein A-IV (1.0%)	Thrombospondin-1 (1.0%)	Inter α-trypsin inhibitor, heavy chain 4 (1.0%)	Isoform 2 of Ig γ-3 chain C region (0.7%)	Ig kappa chain C region (0.3%)	Complement C1q subcomponent subunit A (0.4%)	Complement C1q subcomponent subunit C (0.7%)	Apolipoprotein A-IV (0.9%)	Apolipoprotein E (0.7%)	Apolipoprotein E (1.0%)
**20**	Complement C1q subcomponent subunit B (0.6%)	Ceruloplasmin (0.5%)	Serine protease inhibitor A3K (0.9%)	Apolipoprotein A-IV (0.9%)	Plasminogen (0.9%)	Transitional endoplasmic reticulum ATPase (0.6%)	Ig lambda-2 chain C region (0.3%)	Complement C5 (0.4%)	Apolipoprotein A-I (0.6%)	Murinoglobulin-1 (0.9%)	Complement C1q subcomponent subunit B (0.7%)	Transitional endoplasmic reticulum ATPase (0.9%)
**21**	Complement C4-B (0.6%)	Ficolin-1 (0.5%)	Complement C4-B (0.9%)	Mannose-binding protein C (0.9%)	Complement C1q subcomponent subunit A (0.8%)	Complement C1q subcomponent subunit A (0.6%)	Ig kappa chain V-V region HP 93G7 (0.3%)	Ceruloplasmin (0.4%)	Histidine-rich glycoprotein (0.5%)	FXIII B chain (0.9%)	Complement C1q subcomponent subunit A (0.7%)	Ceruloplasmin (0.9%)
**22**	Complement C1q subcomponent subunit A (0.6%)	Complement C4-B (0.5%)	Clusterin (0.7%)	Clusterin (0.9%)	Serum albumin (0.7%)	Complement C4-B (0.6%)	Isoform 2 of Ig γ-3 chain C region (0.3%)	H-2 class I histocompatibility antigen, TLA(B) α chain (0.4%)	Serum albumin (0.4%)	Complement C4-B (0.8%)	Clusterin (0.6%)	Plasminogen (0.9%)
**23**	Prothrombin (0.4%)	α-2-macroglobulin-P (0.4%)	Apolipoprotein E (0.6%)	Inter α-trypsin inhibitor, heavy chain 4 (0.9%)	Ig µ chain C region secreted form (0.7%)	Histidine-rich glycoprotein (0.5%)	Histidine-rich glycoprotein (0.2%)	Complement C1q subcomponent subunit C (0.4%)	Isoform 2 of Ig γ-3 chain C region (0.4%)	Apolipoprotein C-III (0.8%)	Apolipoprotein C-I (0.5%)	Apolipoprotein C-III (0.8%)
**24**	Catalase (0.4%)	Inter α-trypsin inhibitor, heavy chain 4 (0.4%)	Serum albumin (0.6%)	Inter-α-trypsin inhibitor heavy chain H2 (0.9%)	Apolipoprotein A-II (0.6%)	Serum amyloid P-component (0.3%)	CD5 antigen-like (0.2%)	Ig lambda-2 chain C region (0.4%)	Catalase (0.4%)	Apolipoprotein A-II (0.8%)	Serine protease inhibitor A3K (0.4%)	Complement C1q subcomponent subunit A (0.8%)
**25**	Ficolin-1 (0.4%)	Serotransferrin (0.4%)	Apolipoprotein A-II (0.5%)	Complement C1q subcomponent subunit C (0.8%)	Ficolin-1 (0.6%)	Isoform 2 of Mannan-binding lectin serine protease 1 (0.3%)	Adiponectin (0.2%)	von Willebrand factor (0.4%)	Ficolin-1 (0.3%)	Inter-α-trypsin inhibitor heavy chain H1 (0.7%)	Plasminogen (0.4%)	Clusterin (0.8%)

Supplementary Table 5. The TOP list of proteins presents in protein corona of SMPN-1 after 8, 24, and 48 h in circulation. Proteins are ranked by abundance from highest to lowest. Each column represents proteins identified on a single biological replicate with 4 biological replicates shown per treatment group. Summative data for each treatment group was generated by combining the proteins identified in each biological replicate to a full list of proteins identified on SMPNs collected within the molecular weight group and time point group A complete dataset of proteins is given in Supplementary Data S1.

Supplementary Table 6. Unique proteins on SMPN-1 at 8, 24, and 48 hours			
	8 Hours	24 Hours	48 Hours
1	Galactose-3-O-sulfotransferase 3	MAP7 domain-containing protein 2	Glutamine synthetase
2	Collagen alpha-1(VI) chain	Calpain small subunit 1 (Fragment)	Adiponectin
3	Vitamin D-binding protein		26S proteasome non-ATPase regulatory subunit 1
4	Vitamin K-dependent protein C		26S proteasome non-ATPase regulatory subunit 7
5	Proteasome subunit beta type-6		Ig heavy chain V region PJ14
6	Neuronal acetylcholine receptor subunit alpha-4		Latent-transforming growth factor beta-binding protein 1
7	Hemopexin		60S ribosomal protein L10 (Fragment)
8	Protein 1300017J02Rik		Cytosolic 10-formyltetrahydrofolate dehydrogenase
9	Corticosteroid-binding globulin		HAUS augmin-like complex subunit 1
10	Isoform 2 of Complement factor D		Alpha-crystallin B chain
11	Fructose-biphosphate aldolase (Fragment)		Argininosuccinate synthase
12	Serine protease inhibitor A3N		60S ribosomal protein L3
13	Protein Col6a3		Cytochrome P450 2D10
14	Major urinary protein 8		Haptoglobin
15	Histone H4		Collagen alpha-1(VI) chain
16	Cytochrome P450 2D9		Protein Col6a3
17	Proteasome subunit beta type-4		Protein Ahnak2 (Fragment)
18	Isoform 2 of Oncoprotein-induced transcript 3 protein		
19	Formimidoyltransferase-cyclodeaminase		
20	Proteasome subunit alpha type-4		
21	Major urinary protein 2		
22	Elongation factor 1-delta (Fragment)		
23	Latent-transforming growth factor beta-binding protein 1		
24	Ig alpha chain C region		
25	Ferritin		
26	Proteasome subunit alpha type-3		
27	Extracellular matrix protein 1		
28	Erythrocyte band 7 integral membrane protein		
29	Protein C7		
30	Cytochrome P450 2C29		
31	Mannose-binding protein A		
32	Protein Epb4.1		
33	Beta-2-glycoprotein 1		
34	Ig kappa chain V-V region MOPC 173		
35	Histone H3 (Fragment)		
36	Isoform 2 of Afamin		
37	60S ribosomal protein L35a		
38	Protein C6		
39	Ras-related protein Rap-1b		
40	Alpha-crystallin B chain		
41	Isoform 2 of Spectrin alpha chain, non-erythrocytic		
42	Protein Ahnak2 (Fragment)		
43	Complement component 8, gamma subunit, isoform CRA_b		
44	Coagulation factor X		
45	Titin		
46	Ig lambda-2 chain V region		
47	Actin, alpha skeletal muscle		
48	Integrin beta-3		
49	Tyrosine-protein kinase BAZ1B		
50	ATP synthase subunit beta, mitochondrial		
51	Cofilin-1		
52	Protein Shroom1		

Supplementary Table 6. **Unique proteins identified on SMPN-1 after 8, 24 and 48 h**. List of proteins identified on SMPN-1 after 8, 24, and 48 h. Proteins are ranked by abundance from highest to lowest. Summative data for each treatment group was generated by combining the proteins identified in each biological replicate to a full list of proteins identified on SMPNs collected within the molecular weight group and time point group. A complete dataset of proteins is given in Supplementary Data 1. Protein abundances are not provided here since protein abundances were taken as additive data from 4 different mice; however, the abundance of all individual identified proteins (N = 4 mice) are given in Supplementary Data 1.

Supplementary Table 7. Common proteins between SMPN-1 at 8, 24 and 48 h	
1	Protein Fga
2	Alpha-2-macroglobulin
3	Fibrinogen gamma chain
4	Fibrinogen beta chain
5	Fibronectin
6	Coagulation factor XIII A chain
7	Coagulation factor XIII B chain
8	Complement factor H
9	Apolipoprotein A-I
10	Serum albumin
11	Ig mu chain C region secreted form
12	Alpha-2-macroglobulin-P
13	Gelsolin
14	Mannose-binding protein C
15	Plasminogen
16	von Willebrand factor
17	Transitional endoplasmic reticulum ATPase
18	Histidine-rich glycoprotein
19	Complement C1q subcomponent subunit B
20	Complement C4-B
21	Complement C1q subcomponent subunit A
22	Prothrombin
23	Catalase
24	Ficolin-1
25	Isoform 2 of Ig gamma-3 chain C region
26	Complement C1q subcomponent subunit C
27	Platelet factor 4
28	Ig kappa chain C region
29	Cluterin
30	Complement C5
31	Ceruloplasmin
32	Inter-alpha-trypsin inhibitor heavy chain H2
33	CD5 antigen-like
34	Apolipoprotein E
35	Serine protease inhibitor A3K
36	Alpha-1-antitrypsin 1-2
37	Apolipoprotein A-IV
38	Ig kappa chain V-II region 26-10
39	Murinoglobulin-1
40	Ig kappa chain V-V region HP 93G7
41	Isoform 2 of Mannan-binding lectin serine protease 1
42	Glutathione peroxidase 3
43	Alpha-1-antitrypsin 1-1
44	Alpha-2-antiplasmin
45	Inter alpha-trypsin inhibitor, heavy chain 4
46	Collectin-11
47	Kininogen-1
48	Proteasome subunit alpha type-7
49	Serotransferrin
50	Adiponectin
51	Actin, cytoplasmic 1
52	Proteasome subunit alpha type-6
53	Alpha-2-HS-glycoprotein
54	Serum Amyloid A-1
55	Inter-alpha-trypsin inhibitor heavy chain H1
56	Angiopoietin-related protein 6
57	Serum amyloid P-component
58	Alpha-amylase 1
59	Collectin-10
60	Proteasome subunit alpha type-1
61	Dynactin subunit 2
62	Thrombospondin-1
63	Fatty acid synthase
64	Carboxypeptidase N subunit 2
65	Collagen alpha-1(VI) chain
66	Vitamin D-binding protein
67	Glutamine synthetase
68	Cytosolic 10-formyltetrahydrofolate dehydrogenase
69	Complement component C8 beta chain
70	Carboxypeptidase N catalytic chain
71	Isoform Short of Tripeptidyl-peptidase 2
72	Isoform 2 of Acylamino-acid-releasing enzyme
73	Proteasome subunit beta type-6
74	Ig kappa chain V-V region K2 (Fragment)
75	Protein Gm20547
76	Apolipoprotein A-II
77	Coagulation factor V
78	Hemopexin
79	78 kDa glucose-regulated protein
80	Cytochrome b-5, isoform CRA_a
81	H-2 class I histocompatibility antigen, Q10 alpha chain
82	C-reactive protein
83	Heat shock cognate 71 kDa protein
84	Clathrin heavy chain 1
85	Protein 1300017J02Rik
86	Zinc-alpha-2-glycoprotein
87	Beta-2-microglobulin

Supplementary Table 7. Common proteins between SMPN-1 at 8, 24 and 48 h (cont.).	
88	Ig kappa chain V-II region 7S34.1
89	Isoform 2 of Complement factor D
90	Carboxylesterase 1C
91	Apolipoprotein C-III
92	Cytochrome P450 3A41
93	Immunoglobulin J chain (Fragment)
94	Myosin-9
95	Complement component C8 alpha chain
96	Isoform 3 of Reelin
97	Complement component C9
98	Multimerin-1
99	Apolipoprotein C-IV
100	Spectrin alpha chain, erythrocytic 1
101	Apolipoprotein C-II
102	Ig heavy chain V region M511
103	Major urinary protein 8
104	Spectrin beta 1
105	Band 3 anion transport protein
106	Ig lambda-2 chain C region
107	Apolipoprotein B-100 (Fragment)
108	Angiopoietin-1
109	BPI fold-containing family A member 2 (Fragment)
110	Proteasome subunit beta type-1
111	Serum paraoxonase/arylesterase 1
112	Cytochrome P450 2D10
113	Serum amyloid A-4
114	Proteasome subunit alpha type-5
115	Lysyl oxidase homolog 1
116	Dynactin subunit 1
117	Putative ATP-dependent Clp protease proteolytic subunit, mitochondrial
118	Alpha-centractin
119	Ankyrin-1
120	Uncharacterized protein
121	Thrombospondin-4
122	Proteasome subunit alpha type-2
123	Mannan-binding lectin serine protease 1
124	Proteasome subunit beta type-4
125	Protein Tfg
126	Protein-glutamine gamma-glutamyltransferase 2
127	C4b-binding protein
128	Ig lambda-1 chain C region
129	Major vault protein
130	Myosin light polypeptide 6
131	C-type lectin domain family 11 member A
132	Ig kappa chain V-VI region J539
133	Ig kappa chain V-V region HP 91A3
134	Ig kappa chain V-I S107A
135	Ig heavy chain V region MOPC 104E
136	Coagulation factor VIII
137	Aspartyl aminopeptidase
138	60S ribosomal protein L18
139	Inter-alpha-trypsin inhibitor heavy chain H3
140	Hyaluronan-binding protein 2 (Fragment)
141	Proteasome subunit alpha type-4
142	Isoform 5 of periostin
143	Latent-transforming growth factor beta-binding protein 1
144	Antithrombin-III
145	Complement C1r-A subcomponent
146	Proteasome subunit beta type-5
147	Proteasome subunit alpha type-3
148	Extracellular matrix protein 1
149	Ig kappa chain V-V region MPOC 149
150	T-complex protein 1 subunit alpha
151	Beta-centractin
152	Phosphatidylcholine-sterol acyltransferase
153	Complement C1s-A subcomponent
154	26S proteasome non-ATPase regulatory subunit 2 OS=Mus musculus GN=Psmd2 PE=1 SV=1;tr|J3KMQ2|J3KMQ2_MOUSE Uncharacterized protein
155	Mannose-binding protein A
156	Beta-2-glycoprotein 1
157	Isoform 2 of Dynactin subunit 4
158	Proteasome subunit beta type-8
159	T-complex protein 1 subunit theta
160	Secreted phosphoprotein 24
161	Ig kappa chain V-V region L7 (Fragment)
162	60S ribosomal protein L4
163	Isoform 2 of Afamin
164	T-complex protein 1 subunit delta
165	Isoform 3 of sulfhydryl oxidase 1
166	Apolipoprotein C-I
167	ATP-binding cassette sub-family B member 9
168	von Willebrand factor type A Sushi
169	Katanin p60 ATPase-containing subunit 1
170	Fibulin-1
171	Protein Ahnak2 (Fragment)
172	Apolipoprotein A-V
173	Isoform 2 of Tenascin

Supplementary Table 7. Common proteins between SMPN-1 at 8, 24 and 48 h (cont.).	
174	Heparin cofactor 2
175	Mannan-binding lectin serine protease 2
176	EGF-containing fibulin-like extracellular matrix protein 1
177	Inter-alpha trypsin inhibitor, heavy chain 4
178	BAG family molecular chaperone regulator 2
179	Glycosylphosphatidylinositol specific phospholipase D1
180	Ig kappa chain V-V region MOPC 41
181	Transthyretin
182	Coagulation factor X
183	Peroxiredoxin-2
184	T-complex protein 1 subunit beta
185	Ig heavy chain V region 441
186	26S protease regulatory subunit 7
187	Ig lambda-1 chain V region
188	Ig lambda-2 chain V region
189	MCG5400
190	Ig lambda-3 chain C region
191	Galectin-3-binding protein
192	26S protease regulatory subunit 8
193	Aquaporin-1
194	Protein Ces1b
195	Ig kappa chain V-III region PC 3741
196	Ig heavy chain V region 5-84
197	Complement factor I
198	Phospholipid transfer protein

Supplementary Table 7. Common proteins identified on SMPN-1 between 8, 24, and 48 h. List of common proteins identified on SMPN-1 between 8, 24, and 48 h time points. Proteins are ranked by abundance from highest to lowest. Summative data for each treatment group was generated by combining the proteins identified in each biological replicate to a full list of proteins identified on SMPNs collected within the molecular weight group and time point group. A complete dataset of proteins given in Supplementary Data 1. Protein abundances are not provided here since protein abundances were taken as additive data from 4 different mice; however, the abundance of all individual identified proteins (N = 4 mice) are given in Supplementary Data 1.

Supplementary Table 8. Top 25 proteins on SMPN-1, -3, and -9 8 hours post-injection. Four independent biological replicates are shown.

	SMPN-1 (8 h)				SMPN-3 (8 h)				SMPN-9 (8 h)			
**1**	Protein Fga (21.0%)	Protein Fga (24.1%)	α-2-macroglobulin (18.2%)	Protein Fga (13.5%)	Fibrinogen γ chain (24.6%)	Fibronectin (13.9%)	Protein Fga (21.8%)	Protein Fga (27.6%)	Fibronectin (14.1%)	Protein Fga (28.8%)	α-2-macroglobulin (21.0%)	Protein Fga (30.2%)
**2**	α-2-macroglobulin (18.0%)	α-2-macroglobulin (15.9%)	Protein Fga (11.9%)	Fibrinogen γ chain (11.7%)	Fibrinogen β chain (21.2%)	Gelsolin (8.8%)	Fibrinogen γ chain (13.3%)	Fibrinogen β chain (22.1%)	Gelsolin (12.4%)	Fibrinogen β chain (21.6%)	Protein Fga (15.8%)	Fibrinogen β chain (16.0%)
**3**	Fibrinogen γ chain (12.5%)	Fibrinogen γ chain (13.4%)	Fibrinogen γ chain (10.3%)	Fibronectin (11.3%)	Protein Fga (18.6%)	Protein Fga (8.6%)	Fibrinogen β chain (12.4%)	Fibrinogen γ chain (16.1%)	Protein Fga (10.2%)	α-2-macroglobulin (16.0%)	Serum albumin (14.7%)	Fibrinogen γ chain (15.9%)
**4**	Fibrinogen β chain (9.1%)	Fibrinogen β chain (8.6%)	Fibrinogen β chain (6.8%)	Fibrinogen β chain (10.5%)	Fibronectin (7.0%)	Fibrinogen γ chain (4.7%)	Complement C3 (8.5%)	α-2-macroglobulin (10.3%)	α-2-macroglobulin (9.7%)	Fibrinogen γ chain (15.7%)	Fibronectin (10.2%)	α-2-macroglobulin (9.6%)
**5**	Fibronectin (7.0%)	FXIII A chain (6.2%)	Fibronectin (6.5%)	FXIII A chain (4.9%)	FXIII A chain (4.5%)	Fibrinogen β chain (4.5%)	α-2-macroglobulin (8.0%)	Ig μ chain C region secreted form (4.1%)	Fibrinogen γ chain (6.5%)	Serum albumin (3.9%)	Fibrinogen β chain (7.6%)	FXIII B chain (5.1%)
**6**	FXIII A chain (5.8%)	FXIII B chain (4.4%)	FXIII A chain (4.6%)	Apolipoprotein A-I (4.0%)	FXIII B chain (3.5%)	Transitional endoplasmic reticulum ATPase (4.4%)	Fibronectin (7.8%)	Fibronectin (3.1%)	Fibrinogen β chain (6.3%)	Fibronectin (3.1%)	Fibrinogen γ chain (6.5%)	Ig μ chain C region secreted form (4.8%)
**7**	FXIII B chain (3.9%)	Fibronectin (3.5%)	Ig μ chain C region secreted form (4.0%)	Gelsolin (3.8%)	α-2-macroglobulin (3.1%)	Complement C3 (4.4%)	FXIII A chain (4.2%)	Complement C3 (2.6%)	Apolipoprotein A-I (5.9%)	FXIII A chain (2.4%)	Apolipoprotein A-I (5.7%)	FXIII A chain (3.6%)
**8**	Complement C3 (1.9%)	Serum albumin (2.5%)	Apolipoprotein A-I (3.2%)	α-2-macroglobulin (3.1%)	Complement C3 (2.2%)	Mannose-binding protein C (4.3%)	FXIII B chain (3.4%)	FXIII B chain (2.0%)	Mannose-binding protein C (4.9%)	FXIII B chain (2.4%)	Carbonic anhydrase 2 (4.0%)	Serum albumin (2.1%)
**9**	Apolipoprotein A-I (1.7%)	Apolipoprotein A-I (2.0%)	Transitional endoplasmic reticulum ATPase (2.0%)	von Willebrand factor (2.8%)	Transitional endoplasmic reticulum ATPase (2.1%)	Apolipoprotein A-I (4.1%)	Mannose-binding protein C (2.9%)	FXIII A chain (1.5%)	Inter-α-trypsin inhibitor heavy chain H1 (4.1%)	Ig μ chain C region secreted form (1.9%)	Complement C3 (3.0%)	Fibronectin (2.1%)
**10**	Serum albumin (1.6%)	Complement C3 (1.7%)	Complement C1q subcomponent subunit B (2.0%)	Complement C4-B (2.8%)	Ig μ chain C region secreted form (1.5%)	α-2-macroglobulin (3.7%)	Ig μ chain C region secreted form (2.8%)	Transitional endoplasmic reticulum ATPase (1.2%)	Complement C3 (2.7%)	Apolipoprotein A-I (1.8%)	Gelsolin (2.7%)	Apolipoprotein A-I (1.2%)
**11**	Ig μ chain C region secreted form (1.4%)	Gelsolin (1.5%)	Complement C3 (1.8%)	Apolipoprotein E (1.4%)	Plasminogen (1.2%)	Complement C1q subcomponent subunit B (2.6%)	Apolipoprotein A-I (2.1%)	Serum albumin (1.0%)	Complement C4-B (2.3%)	Complement C3 (0.9%)	α-1-antitrypsin 1-2 (2.3%)	Immunoglobulin J chain (Fragment) (1.0%)
**12**	α-2-macroglobulin-P (1.1%)	Transitional endoplasmic reticulum ATPase (1.3%)	FXIII B chain (1.8%)	Transitional endoplasmic reticulum ATPase (1.4%)	Mannose-binding protein C (1.1%)	Apolipoprotein A-IV (2.3%)	Complement C1q subcomponent subunit B (1.8%)	Ig k chain V-V region HP 93G7 (0.6%)	Inter-α-trypsin inhibitor heavy chain H2 (2.3%)	Gelsolin (0.7%)	Ig μ chain C region secreted form (1.6%)	Complement C3 (0.9%)
**13**	Gelsolin (1.0%)	Ig μ chain C region secreted form (1.0%)	Complement C1q subcomponent subunit A (1.6%)	Ig μ chain C region secreted form (1.3%)	Complement C1q subcomponent subunit B (0.8%)	Inter-α-trypsin inhibitor heavy chain H1 (2.1%)	Transitional endoplasmic reticulum ATPase (1.7%)	Complement C1q subcomponent subunit B (0.6%)	Inter α-trypsin inhibitor, heavy chain 4 (1.7%)	Mannose-binding protein C (0.7%)	FXIII B chain (0.9%)	Ig λ-2 chain C region (0.7%)
**14**	Mannose-binding protein C (1.0%)	Plasminogen (0.8%)	Gelsolin (1.4%)	Complement C3 (1.3%)	Serum albumin (0.7%)	Complement C4-B (1.7%)	Serum albumin (1.3%)	Apolipoprotein A-I (0.6%)	von Willebrand factor (1.4%)	Protein Ces1b (0.1%)	Carbonic anhydrase 1 (0.9%)	Ig k chain C region (0.5%)
**15**	Plasminogen (0.9%)	Complement C1q subcomponent subunit B (0.8%)	Murinoglobulin-1 (1.3%)	Complement C1q subcomponent subunit B (1.1%)	Complement C1q subcomponent subunit C (0.7%)	Complement C1q subcomponent subunit A (1.6%)	Gelsolin (1.2%)	Mannose-binding protein C (0.5%)	Serum albumin (1.1%)	Serum amyloid P-component (<0.1%)	Serotransferrin (0.8%)	Gelsolin (0.4%)

Supplementary Table 9. Top 25 proteins on SMPN-1, -3, and -9 8 hours post-injection. Four independent biological replicates are shown (cont.).

	SMPN-1 (8 h)				SMPN-3 (8 h)				SMPN-9 (8 h)			
**16**	von Willebrand factor (0.8%)	Mannose-binding protein C (0.6%)	Complement C1q subcomponent subunit C (1.1%)	Inter-α-trypsin inhibitor heavy chain H1 (1.1%)	Apolipoprotein A-I (0.6%)	Ig µ chain C region secreted form (1.6%)	Complement C1q subcomponent subunit A (1.2%)	Ig k chain C region (0.5%)	FXIII A chain (0.9%)		Apolipoprotein A-II (0.7%)	α-1-antitrypsin 1-2 (0.4%)
**17**	Transitional endoplasmic reticulum ATPase (0.8%)	Serine protease inhibitor A3K (0.6%)	von Willebrand factor (1.1%)	FXIII B chain (1.0%)	Histidine-rich glycoprotein (0.6%)	Inter-α-trypsin inhibitor heavy chain H2 (1.6%)	von Willebrand factor (0.9%)	α-amylase 1 (0.4%)	Clusterin (0.8%)		Peroxiredoxin-2 (0.5%)	Murinoglobulin-1 (0.4%)
**18**	Galactose-3-O-sulfotransferase 3 (0.8%)	Complement C1q subcomponent subunit C (0.6%)	Plasminogen (1.0%)	Complement C1q subcomponent subunit A (1.0%)	Complement C1q subcomponent subunit A (0.6%)	FXIII A chain (1.3%)	Actin, cytoplasmic 1 (0.6%)	Immunoglobulin J chain (Fragment) (0.4%)	Apolipoprotein A-II (0.8%)		α-1-antitrypsin 1-1 (0.5%)	Ig k chain V-II region 26-10 (0.4%)
**19**	Histidine-rich glycoprotein (0.7%)	Complement C1q subcomponent subunit A (0.6%)	Apolipoprotein A-IV (1.0%)	Thrombospondin-1 (1.0%)	Ficolin-1 (0.5%)	Complement C1q subcomponent subunit C (1.2%)	Histidine-rich glycoprotein (0.5%)	Putative ATP-dependent Clp protease proteolytic subunit, mitochondrial (0.4%)	Actin, cytoplasmic 1 (0.8%)		Band 3 anion transport protein (0.3%)	Histidine-rich glycoprotein (0.4%)
**20**	Complement C1q subcomponent subunit B (0.6%)	Ceruloplasmin (0.5%)	Serine protease inhibitor A3K (0.9%)	Apolipoprotein A-IV (0.9%)	Complement C4-B (0.5%)	Thrombospondin-1 (1.1%)	Complement C1q subcomponent subunit C (0.5%)	Complement C1q subcomponent subunit A (0.4%)	Inter-α-trypsin inhibitor heavy chain H3 (0.8%)		Isoform 2 of Probably ATP-dependent RNA helicase DDX17 (0.2%)	Ig heavy chain V region MOPC 104E (0.3%)
**21**	Complement C4-B (0.6%)	Ficolin-1 (0.5%)	Complement C4-B (0.9%)	Mannose-binding protein C (0.9%)	von Willebrand factor (0.4%)	von Willebrand factor (1.0%)	Plasminogen (0.4%)	H-2 class I histocompatibility antigen, TLA(B) α chain (0.3%)	Apolipoprotein E (0.7%)		Isoform 2 of α-synuclein (0.1%)	H-2 class I histocompatibility antigen, TLA(B) α chain (0.3%)
**22**	Complement C1q subcomponent subunit A (0.6%)	Complement C4-B (0.5%)	Clusterin (0.7%)	Clusterin (0.9%)	CD5 antigen-like (0.4%)	Inter α-trypsin inhibitor, heavy chain 4 (0.9%)	Carbonic anhydrase 2 (0.3%)	CD5 antigen-like (0.3%)	Prothrombin (0.7%)		Ankyrin-1 (0.1%)	Cytochrome P450 3A41 (0.3%)
**23**	Prothrombin (0.4%)	α-2-macroglobulin-P (0.4%)	Apolipoprotein E (0.6%)	Inter α-trypsin inhibitor, heavy chain 4 (0.9%)	Gelsolin (0.4%)	Clusterin (0.8%)	Platelet factor 4 (0.3%)	Complement C4-B (0.2%)	Ig µ chain C region secreted form (0.7%)			Ig k chain V-V region HP 93G7 (0.3%)
**24**	Catalase (0.4%)	Inter α-trypsin inhibitor, heavy chain 4 (0.4%)	Serum albumin (0.6%)	Inter-α-trypsin inhibitor heavy chain H2 (0.9%)	Thrombospondin-1 (0.3%)	Isoform 2 of Mannan-binding lectin serine protease 1 (0.8%)	Complement C5 (0.3%)	Platelet factor 4 (0.2%)	Complement C1q subcomponent subunit A (0.6%)			Mannose-binding protein C (0.3%)
**25**	Ficolin-1 (0.4%)	Serotransferrin (0.4%)	Apolipoprotein A-II (0.5%)	Complement C1q subcomponent subunit C (0.8%)	Platelet factor 4 (0.2%)	Actin, cytoplasmic 1 (0.8%)	Complement C4-B (0.2%)	Ig λ-2 chain C region	α-2-HS-glycoprotein (0.6%)			Putative ATP-dependent Clp protease proteolytic subunit, mitochondrial (0.3%)

Supplementary Table 8. List of all proteins on SMPN-1, 3, and 9 after 8 h circulation *in vivo*. Proteins are ranked by abundance from highest to lowest with relative abundance (%) given for >1%. Summative data for each treatment group was generated by combining the proteins identified in each biological replicate to a full list of proteins identified on SMPNs collected within the molecular weight group and time point group. A complete dataset of proteins is given in Supplementary Data 1.

Supplementary Table 9. Top 25 proteins on SMPN-1, -3, and -9, 48 hours post-injection. Four independent biological replicates are shown.

	SMPN-1 (48 h)				SMPN-3 (48 h)				SMPN-9 (48 h)			
**1**	α-2-macroglobulin (18.4%)	α-2-macroglobulin (26.0%)	α-2-macroglobulin (21.7%)	α-2-macroglobulin (20.2%)	α-2-macroglobulin (18.5%)	α-2-macroglobulin (30.5%)	Protein Fga (9.5%)	α-2-macroglobulin (17.9%)	Protein Fga (36.6%)	α-2-macroglobulin (29.0%)	α-2-macroglobulin (21.7%)	Protein Fga (29.3%)
**2**	Fibrinogen γ chain (17.0%)	Protein Fga (9.2%)	Protein Fga (15.9%)	Fibrinogen γ chain (9.4%)	Protein Fga (8.8%)	Protein Fga (6.5%)	Fibronectin (8.3%)	Protein Fga (10.0%)	Fibrinogen β chain (19.2%)	Protein Fga (21.9%)	Fibronectin (11.8%)	Fibrinogen β chain (19.3%)
**3**	Protein Fga (15.6%)	Fibrinogen γ chain (8.9%)	Fibrinogen γ chain (11.3%)	Protein Fga (9.2%)	Fibronectin (8.4%)	Fibronectin (6.5%)	Fibrinogen γ chain (6.0%)	Complement C3 (8.4%)	Fibrinogen γ chain (17.9%)	Fibrinogen γ chain (10.3%)	Protein Fga (10.0%)	Fibrinogen γ chain (15.8%)
**4**	FXIII A chain (9.2%)	Fibronectin (5.2%)	Fibronectin (8.9%)	Fibronectin (6.5%)	Fibrinogen γ chain (6.3%)	Complement C3 (5.7%)	Apolipoprotein A-I (5.3%)	Fibronectin (8.2%)	α-2-macroglobulin (7.6%)	Fibrinogen β chain (10.2%)	Gelsolin (5.9%)	α-2-macroglobulin (11.9%)
**5**	Fibrinogen β chain (7.3%)	Fibrinogen β chain (5.2%)	Fibrinogen β chain (7.8%)	Fibrinogen β chain (5.5%)	Apolipoprotein A-I (5.2%)	Gelsolin (4.4%)	Fibrinogen β chain (5.0%)	Fibrinogen γ chain (7.2%)	Ig µ chain C region secreted form (5.2%)	Titin (4.8%)	Apolipoprotein A-IV (5.3%)	Ig µ chain C region secreted form (5.1%)
**6**	FXIII B chain (5.2%)	Apolipoprotein A-I (3.9%)	Apolipoprotein A-IV (3.0%)	FXIII A chain (3.6%)	Gelsolin (5.1%)	Apolipoprotein A-I (3.8%)	α-2-macroglobulin (4.6%)	Gelsolin (3.9%)	Serum albumin (3.0%)	Carbonic anhydrase 2 (3.2%)	Apolipoprotein A-I (4.3%)	FXIII B chain (2.6%)
**7**	Fibronectin (3.3%)	Gelsolin (3.2%)	Gelsolin (2.6%)	Ig µ chain C region secreted form (3.4%)	Complement C3 (4.4%)	Fibrinogen γ chain (3.2%)	Apolipoprotein A-IV (4.3%)	Fibrinogen β chain (3.8%)	FXIII B chain (1.7%)	Ig µ chain C region secreted form (3.0%)	Fibrinogen γ chain (4.1%)	FXIII A chain (2.1%)
**8**	Transitional endoplasmic reticulum ATPase (2.6%)	Complement C3 (3.1%)	Apolipoprotein A-I (2.2%)	Apolipoprotein A-I (3.2%)	Fibrinogen β chain (3.8%)	Ig µ chain C region secreted form (3.2%)	Gelsolin (3.6%)	FXIII A chain (2.8%)	Transitional endoplasmic reticulum ATPase (1.3%)	Serum albumin (2.4%)	Fibrinogen β chain (3.9%)	Apolipoprotein A-I (1.9%)
**9**	Ig µ chain C region secreted form (2.0%)	FXIII A chain (2.2%)	FXIII A chain (2.2%)	Complement C3 (2.6%)	Mannose-binding protein C (2.6%)	FXIII A chain (2.4%)	Complement C3 (3.0%)	Apolipoprotein A-IV (2.7%)	FXIII A chain (1.1%)	Fibronectin (2.3%)	Complement C3 (2.7%)	Serum albumin (1.8%)
**10**	Mannose-binding protein C (1.4%)	Serum albumin (2.1%)	Mannose-binding protein C (2.0%)	Gelsolin (2.3%)	FXIII A chain (1.8%)	Fibrinogen β chain (2.1%)	Serum albumin (2.4%)	Apolipoprotein A-I (2.4%)	Apolipoprotein A-I (1.1%)	Complement C3 (2.2%)	Complement C4-B (2.2%)	Fibronectin (1.4%)
**11**	Complement C3 (1.4%)	Ceruloplasmin (1.9%)	Complement C3 (1.9%)	Complement C4-B (1.3%)	Apolipoprotein A-IV (1.8%)	Mannose-binding protein C (1.4%)	FXIII A chain (2.3%)	Complement C4-B (2.4%)	Cytochrome P450 3A41 (1.0%)	FXIII A chain (2.2%)	FXIII A chain (2.1%)	Complement C3 (1.3%)
**12**	Gelsolin (1.3%)	Ig µ chain C region secreted form (1.7%)	Transitional endoplasmic reticulum ATPase (1.4%)	Apolipoprotein A-IV (1.2%)	Serum albumin (1.6%)	von Willebrand factor (1.4%)	Myosin-9 (2.3%)	Ig µ chain C region secreted form (2.3%)	Ig kappa chain V-V region HP 93G7 (0.5%)	FXIII B chain (1.2%)	Mannose-binding protein C (1.4%)	Tripartite motif-containing protein 60 (0.6%)
**13**	Complement C1q subcomponent subunit B (1.2%)	Mannose-binding protein C (1.4%)	Ig µ chain C region secreted form (0.9%)	Murinoglobulin-1 (1.2%)	Apolipoprotein C-III (1.5%)	Transitional endoplasmic reticulum ATPase (1.4%)	Actin, cytoplasmic 1 (2.2%)	von Willebrand factor (1.3%)	Complement C3 (0.5%)	Apolipoprotein A-I (0.8%)	von Willebrand factor (1.4%)	Complement C1q subcomponent subunit B (0.6%)
**14**	von Willebrand factor (1.2%)	Apolipoprotein C-I (1.1%)	Isoform 7 of Nuclear factor 1 C-type (0.9%)	Serum albumin (1.2%)	Apolipoprotein A-II (1.5%)	Apolipoprotein A-IV (1.3%)	von Willebrand factor (1.7%)	FXIII B chain (1.2%)	Complement C1q subcomponent subunit B (0.5%)	Transitional endoplasmic reticulum ATPase (0.6%)	Complement C1q subcomponent subunit B (1.3%)	Immunoglobulin J chain (Fragment) (0.5%)
**15**	Plasminogen (1.0%)	Plasminogen (1.1%)	Serum albumin (0.8%)	FXIII B chain (1.1%)	Ceruloplasmin (1.5%)	Apolipoprotein C-III (1.2%)	Apolipoprotein C-III (1.7%)	Complement C1q subcomponent subunit B (1.2%)	Ig lambda-2 chain C region (0.5%)	Mannose-binding protein C (0.5%)	Serum albumin (1.2%)	α-1-antitrypsin 1-2 (0.5%)

Supplementary Table 9. Top 25 proteins on SMPN-1, -3, and -9, 48 hours post-injection. Four independent biological replicates are shown (cont.).

	SMPN-1 (48 h)				SMPN-3 (48 h)				SMPN-9 (48 h)			
**16**	α-2-macroglobulin-P (0.9%)	Apolipoprotein E (1.0%)	Apolipoprotein C-III (0.8%)	Complement C1q subcomponent subunit B (1.1%)	von Willebrand factor (1.4%)	Murinoglobulin-1 (1.2%)	Ig μ chain C region secreted form (1.6%)	Complement C1q subcomponent subunit A (1.1%)	Serotransferrin (0.4%)	Carbonic anhydrase 1 (0.5%)	Major urinary protein 8 (1.2%)	Ig lambda-2 chain C region (0.4%)
**17**	Complement C4-B (0.9%)	Inter-α-trypsin inhibitor heavy chain H2 (0.9%)	Complement C4-B (0.8%)	von Willebrand factor (1.1%)	Inter-α-trypsin inhibitor heavy chain H2 (1.2%)	FXIII B chain (1.1%)	Transitional endoplasmic reticulum ATPase (1.5%)	Murinoglobulin-1 (1.0%)	Fibronectin (0.4%)	Serine protease inhibitor A3K (0.4%)	Transitional endoplasmic reticulum ATPase (0.9%)	Transitional endoplasmic reticulum ATPase (0.4%)
**18**	Complement C1q subcomponent subunit A (0.8%)	von Willebrand factor (0.9%)	FXIII B chain (0.8%)	Mannose-binding protein C (1.0%)	Ig μ chain C region secreted form (1.2%)	Serum albumin (0.9%)	Mannose-binding protein C (1.3%)	Apolipoprotein E (1.0%)	Putative ATP-dependent Clp protease proteolytic subunit, mitochondrial (0.4%)	Ceruloplasmin (0.4%)	Complement C1q subcomponent subunit A (0.9%)	Murinoglobulin-1 (0.4%)
**19**	Complement C1q subcomponent subunit C (0.7%)	Apolipoprotein A-IV (0.9%)	Apolipoprotein E (0.7%)	Apolipoprotein E (1.0%)	Transitional endoplasmic reticulum ATPase (1.2%)	Ceruloplasmin (0.9%)	Complement C4-B (1.2%)	Mannose-binding protein C (1.0%)	Complement C1q subcomponent subunit A (0.3%)	Peroxiredoxin-2 (0.4%)	Inter-α-trypsin inhibitor heavy chain H1 (0.8%)	Complement C1q subcomponent subunit A (0.3%)
**20**	Apolipoprotein A-I (0.6%)	Murinoglobulin-1 (0.9%)	Complement C1q subcomponent subunit B (0.7%)	Transitional endoplasmic reticulum ATPase (0.9%)	Complement C4-B (1.1%)	Inter-α-trypsin inhibitor heavy chain H2 (0.9%)	Thrombospondin-1 (1.1%)	Apolipoprotein C-III (1.0%)	Mannose-binding protein C (0.3%)	Complement C1q subcomponent subunit A (0.4%)	Ig μ chain C region secreted form (0.8%)	α-1-antitrypsin 1-1 (0.3%)
**21**	Histidine-rich glycoprotein (0.5%)	FXIII B chain (0.9%)	Complement C1q subcomponent subunit A (0.7%)	Ceruloplasmin (0.9%)	Plasminogen (0.9%)	Complement C1q subcomponent subunit A (0.9%)	Apolipoprotein E (1.0%)	Clusterin (0.9%)	Ficolin-1 (0.2%)	Complement C1q subcomponent subunit B (0.4%)	FXIII B chain (0.7%)	Serine protease inhibitor A3K (0.3%)
**22**	Serum albumin (0.4%)	Complement C4-B (0.8%)	Clusterin (0.6%)	Plasminogen (0.9%)	Apolipoprotein E (0.9%)	Complement C1q subcomponent subunit B (0.8%)	Inter-α-trypsin inhibitor heavy chain H2 (1.0%)	Transitional endoplasmic reticulum ATPase (0.9%)	Ig kappa chain C region (0.2%)	Neuronal acetylcholine receptor subunit α-4 (0.3%)	Apolipoprotein A-II (0.7%)	Complement C1q subcomponent subunit C (0.3%)
**23**	Isoform 2 of Ig γ-3 chain C region (0.4%)	Apolipoprotein C-III (0.8%)	Apolipoprotein C-I (0.5%)	Apolipoprotein C-III (0.9%)	Inter-α-trypsin inhibitor heavy chain H1 (0.9%)	Complement C4-B (0.8%)	Filamin, α (Fragment) (1.0%)	Complement C1q subcomponent subunit C (0.7%)	Mannose-binding protein A (<0.1%)	α-1-antitrypsin 1-2 (0.3%)	Complement C1q subcomponent subunit C (0.6%)	Serotransferrin (0.3%)
**24**	Catalase (0.4%)	Apolipoprotein A-II (0.8%)	Serine protease inhibitor A3K (0.4%)	Complement C1q subcomponent subunit A (0.8%)	Murinoglobulin-1 (0.8%)	Cytochrome P450 3A41 (0.7%)	Spectrin α chain, erythrocytic 1 (0.9%)	Inter-α-trypsin inhibitor heavy chain H2 (0.7%)		Ig kappa chain V-II region 26-10 (0.2%)	Inter-α-trypsin inhibitor heavy chain H2 (0.6%)	Complement C4-B (0.3%)
**25**	Ficolin-1 (0.3%)	Inter-α-trypsin inhibitor heavy chain H1 (0.7%)	Plasminogen (0.4%)	Clusterin (0.8%)	Angiopoietin-related protein 6 (0.8%)	Apolipoprotein E (0.7%)	Complement C1q subcomponent subunit C (0.9%)	Inter-α-trypsin inhibitor heavy chain H1 (0.6%)		Serum amyloid P-component (0.2%)	Murinoglobulin-1 (0.6%)	Gelsolin (0.3%)

Supplementary Table 9. List of all proteins identified on SMPN-1, 3, and 9 48 hours post-injection. Proteins are ranked by abundance from highest to lowest. Each column represents a biological replicate with data from 4 biological replicates shown per group. Summative data for each treatment group was generated by combining the proteins identified in each biological replicate to a full list of proteins identified on SMPNs collected within the molecular weight group and time point group. A complete dataset of proteins is given in Supplementary Data 1.

Supplementary Table 10. Common proteins between SMPN-1, -3 and -9 at 8 and 48 h		
	**8 Hours**	**48 Hours**
**1**	Protein Fga	Alpha-2-macroglobulin
**2**	Alpha-2-macroglobulin	Fibrinogen gamma chain
**3**	Fibrinogen gamma chain	Protein Fga
**4**	Fibrinogen beta chain	Coagulation factor XIII A chain
**5**	Fibronectin	Fibrinogen beta chain
**6**	Coagulation factor XIII A chain	Coagulation factor XIII B chain
**7**	Coagulation factor XIII B chain	Fibronectin
**8**	Complement factor H	Transitional endoplasmic reticulum ATPase
**9**	Apolipoprotein A-I	Ig mu chain C region secreted form
**10**	Serum albumin	Mannose-binding protein C
**11**	Ig mu chain C region secreted form	Complement factor H
**12**	Gelsolin	Gelsolin
**13**	Mannose-binding protein C	Complement C1q subcomponent subunit B
**14**	Plasminogen	von Willebrand factor
**15**	von Willebrand factor	Plasminogen
**16**	Transitional endoplasmic reticulum ATPase	Alpha-2-macroglobulin-P
**17**	Histidine-rich glycoprotein	Complement C4-B
**18**	Complement C1q subcomponent subunit B	Complement C1q subcomponent subunit A
**19**	Complement C4-B	Complement C1q subcomponent subunit C
**20**	Complement C1q subcomponent subunit A	Apolipoprotein A-I
**21**	Prothrombin	Histidine-rich glycoprotein
**22**	Ficolin-1	Serum albumin
**23**	Isoform 2 of Ig gamma-3 chain C region	Isoform 2 of Ig gamma-3 chain C region
**24**	Complement C1q subcomponent subunit C	Catalase
**25**	Platelet factor 4	Ficolin-1
**26**	Ig kappa chain C region	Cluterin
**27**	Cluterin	Ig kappa chain C region
**28**	Ceruloplasmin	Isoform 2 of Mannan-binding lectin serine protease 1
**29**	Inter-alpha-trypsin inhibitor heavy chain H2	Ig kappa chain V-II region 26-10
**30**	CD5 antigen-like	Glutathione peroxidase 3
**31**	Apolipoprotein E	Alpha-amylase 1
**32**	Serine protease inhibitor A3K	Apolipoprotein E
**33**	Alpha-1-antitrypsin 1-2	Collectin-11
**34**	Apolipoprotein A-IV	Actin, cytoplasmic 1
**35**	Ig kappa chain V-II region 26-10	Ig lambda-2 chain C region
**36**	Murinoglobulin-1	Inter-alpha-trypsin inhibitor heavy chain H2
**37**	Ig kappa chain V-V region HP 93G7	Complement C5
**38**	Isoform 2 of Mannan-binding lectin serine protease 1	Angiopoietin-related protein 6
**39**	Glutathione peroxidase 3	Ig kappa chain V-V region HP 93G7
**40**	Alpha-1-antitrypsin 1-1	Carbonic anhydrase 2
**41**	Alpha-2-antiplasmin	Proteasome subunit alpha type-7
**42**	Inter alpha-trypsin inhibitor, heavy chain 4	Ceruloplasmin
**43**	Collectin-11	Dynactin subunit 2
**44**	Kininogen-1	Proteasome subunit alpha type-6
**45**	Proteasome subunit alpha type-7	Apolipoprotein A-IV
**46**	Serotransferrin	Prothrombin
**47**	Actin, cytoplasmic 1	Thrombospondin-1
**48**	Proteasome subunit alpha type-6	Dynactin subunit 1
**49**	Alpha-2-HS-glycoprotein	Inter-alpha-trypsin inhibitor heavy chain H1
**50**	Inter-alpha-trypsin inhibitor heavy chain H1	Proteasome subunit beta type-1
**51**	Angiopoietin-related protein 6	Apolipoprotein A-II
**52**	Serum amyloid P-component	Proteasome subunit alpha type-2
**53**	Collectin-10	Proteasome subunit alpha type-5
**54**	Thrombospondin-1	Lysyl oxidase homolog 1
**55**	Carboxypeptidase N subunit 2	Platelet factor 4
**56**	Isoform 2 of Acylamino-acid-releasing enzyme	Kininogen-1
**57**	Protein Gm20547	Isoform Short of Tripeptidyl-peptidase 2
**58**	Apolipoprotein A-II	Murinoglobulin-1
**59**	Carbonic anhydrase 2	Proteasome subunit alpha type-1
**60**	H-2 class I histocompatibility antigen, Q10 alpha chain	Heat shock cognate 71 kDa protein
**61**	Heat shock cognate 71 kDa protein	Alpha-1-antitrypsin 1-1
**62**	Ig kappa chain V-II region 7S34.1	Alpha-2-antiplasmin
**63**	Apolipoprotein C-III	Ig heavy chain V region M511
**64**	Cytochrome P450 3A41	Protein-glutamine gamma-glutamyltransferase 2
**65**	Immunoglobulin J chain (Fragment)	Serum amyloid P-component
**66**	Myosin-9	Alpha-2-HS-glycoprotein
**67**	Complement component C8 alpha chain	Collectin-10
**68**	Complement component C9	Proteasome subunit beta type-5
**69**	Apolipoprotein C-IV	Beta-2-microglobulin
**70**	Spectrin alpha chain, erythrocytic 1	Ig heavy chain V region MOPC 104E
**71**	Apolipoprotein C-II	Coagulation factor V
**72**	Ig heavy chain V region M511	Mannan-binding lectin serine protease 1
**73**	Spectrin beta 1	Carbonic anhydrase 1
**74**	Band 3 anion transport protein	Myosin-9
**75**	Ig lambda-2 chain C region	Ig kappa chain V-II region 7S34.1
**76**	Apolipoprotein B-100 (Fragment)	Alpha-centractin
**77**	Proteasome subunit beta type-1	Proteasome subunit beta type-6
**78**	Serum paraoxonase/arylesterase 1	Protein Gm20547
**79**	Serum amyloid A-4	26S proteasome non-ATPase regulatory subunit 2
**80**	Lysyl oxidase homolog 1	Isoform 2 of Acylamino-acid-releasing enzyme
**81**	Putative ATP-dependent Clp protease proteolytic subunit, mitochondrial	Ig lambda-3 chain C region
**82**	Ankyrin-1	Adenylyl cyclase-associated protein 1

Supplementary Table 10. Common Proteins between SMPN-1, -3 and -9 at 8 and 48 h (cont.).		
	8 Hours	48 Hours
83	Uncharacterized protein	26S proteasome non-ATPase regulatory subunit 3
84	Protein-glutamine gamma-glutamyltransferase 2	Apolipoprotein C-I
85	C-type lectin domain family 11 member A	Apolipoprotein C-III
86	Ig kappa chain V-V region HP 91A3	Inter alpha-trypsin inhibitor, heavy chain 4
87	Cathelin-related antimicrobial peptide	Serotransferrin
88	Ig heavy chain V region MOPC 104E	Serine protease inhibitor A3K
89	Inter-alpha-trypsin inhibitor heavy chain H3	Alpha-1-antitrypsin 1-2
90	Isoform 5 of periostin	Carboxypeptidase N subunit 2
91	Phosphatidylcholine-sterol acyltransferase	Carboxypeptidase N catalytic chain
92	T-complex protein 1 subunit theta	Apolipoprotein C-II
93	Isoform 3 of sulfhydryl oxidase 1	Glycosylphosphatidylinositol specific phospholipase D1
94	Lipopolysaccharide-binding protein musculus	H-2 class I histocompatibility antigen, TLA(B) alpha chain
95	Apolipoprotein C-I	Isoform 3 of Reelin
96	Keratin, type II cytoskeletal 2 oral	Vitamin D-binding protein
97	EGF-containing fibulin-like extracellular matrix protein 1	Complement component C8 alpha chain
98	BAG family molecular chaperone regulator 2	Apolipoprotein C-IV
99	Glycosylphosphatidylinositol specific phospholipase D1	H-2 class I histocompatibility antigen, Q10 alpha chain
100	Transthyretin	Serum paraoxonase/arylesterase 1
101	Peroxiredoxin-2	Uncharacterized protein
102	Isoform 2 of Alpha-synuclein	Inter-alpha-trypsin inhibitor heavy chain H3
103	Platelet-activating factor acetylhydrolase	Multimerin-1
104	Ig heavy chain V region 441	Transthyretin
105	Ig lambda-1 chain V region	Coagulation factor VIII
106	Protein Ces1b	Protein 1300017J02Rik
107	T-complex protein 1 subunit gamma	Thrombospondin-4
108		Serum amyloid A-4
109		EGF-containing fibulin-like extracellular matrix protein 1
110		Apolipoprotein B-100 (Fragment)
111		Immunoglobulin J chain (Fragment)
112		Histone H2A type 1-H
113		Zinc-alpha-2-glycoprotein
114		Extracellular matrix protein 1
115		Histone H2B type 1-P
116		Complement component C8 beta chain
117		C4b-binding protein
118		Inter-alpha trypsin inhibitor, heavy chain 4
119		Mannose-binding protein A
120		Peroxiredoxin-2
121		Complement factor I
122		Histone H4
123		Isoform 3 of sulfhydryl oxidase 1
124		Angiopoietin-1
125		Ig kappa chain V-I S107A
126		Coagulation factor X
127		Protein Tfg
128		Isoform 2 of Alpha-synuclein
129		Isoform 5 of periostin
130		Phosphatidylcholine-sterol acyltransferase
131		Ig kappa chain V-V region HP 91A3
132		Mannan-binding lectin serine protease 2
133		Clathrin heavy chain 1
134		60S ribosomal protein L6
135		Major vault protein
136		C-type lectin domain family 11 member A
137		Carboxylesterase 1C
138		Beta-centractin
139		Serum Amyloid A-1
140		Isoform 2 of Dynactin subunit 4
141		BAG family molecular chaperone regulator 2
142		T-complex protein 1 subunit alpha
143		Pigment epithelium-derived factor
144		von Willebrand factor type A Sushi
145		Complement component C9
146		60S ribosomal protein L14
147		Peptidyl-prolyl cis-trans isomerase A
148		T-complex protein 1 subunit delta
149		T-complex protein 1 subunit zeta
150		T-complex protein 1 subunit theta
151		Spectrin alpha chain, erythrocytic 1
152		ATP synthase subunit alpha
153		C-reactive protein
154		Putative ATP-dependent Clp protease proteolytic subunit, mitochondrial
155		Cathelin-related antimicrobial peptide
156		26S protease regulatory subunit 8 OS=Mus musculus GN=Psmc5 PE=2 SV=1;sp|P62196|PRS8_MOUSE 26S protease regulatory subunit 8
157		Alpha-enolase
158		Cytochrome P450 3A41
159		Major urinary protein 8
160		CD5 antigen-like

Supplementary Table 10. Common proteins between SMPN-1, -3, and -9 at 8 and 48 hours (cont.).	
8 Hours	48 Hours
161	Ig lambda-1 chain V region
162	Katanin p60 ATPase-containing subunit 1
163	Ig kappa chain V-III region PC 3741
164	Vitronectin
165	Secreted phosphoprotein 24
166	Histone H3 (Fragment)
167	60S ribosomal protein L18
168	T-complex protein 1 subunit beta
169	Protein Ces1b
170	ATP-binding cassette sub-family B member 9
171	Transferrin receptor protein 1
172	Protein C6
173	Vitamin K-dependent protein C
174	Band 3 anion transport protein
175	Ig heavy chain V region 441
176	Spectrin beta 1
177	Ig kappa chain V-VI region J539
178	Galectin-3-binding protein
179	T-complex protein 1 subunit gamma
180	Annexin A7
181	Ig heavy chain V region 5-84
182	Phospholipid transfer protein
183	Ankyrin-1
184	Fibulin-1
185	Integrin alpha-6

Supplementary Table 10. List of all common proteins identified on SMPN-1, -3 and -9. Data is combined for four mice per group after 8 and 48 h *in vivo*. Proteins are ranked by abundance from highest to lowest. A complete dataset of proteins is given in Supplementary Data 1. Summative data was obtained by combining proteins identified from 4 biological replicates to generate a list of all proteins identified within each SMPN group. Protein abundances are not provided here since protein abundances were taken as additive data from 4 different mice; however, the abundance of all individual identified proteins (N = 4 mice) are given in Supplementary Data 1.

Supplementary Table 11. Unique proteins on SMPN-1, -3, and -9 at 8 and 48 Hours						
	8 Hours			48 hours		
	SMPN-1	SMPN-3	SMPN-9	SMPN-1	SMPN-3	SMPN-9
1	Galactose-3-O-sulfotransferase 3	MAP7 domain-containing protein 2	Isoform 2 of Probably ATP-dependent RNA helicase DDX17	Desmoplakin	Neuronal acetylcholine receptor subunit alpha-4	
2	Collagen alpha-1(VI) chain	Inosine-5-monophosphate dehydrogenase 2	Haptoglobin	55 kDa erythrocyte membrane protein	Lysozyme C-2	
3	Vitamin D-binding protein	T-complex protein 1 subunit eta		Serine protease inhibitor A3G	Proteasome subunit beta type-3	
4	Vitamin K-dependent protein C	Isoform LMW of Kininogen-1		Ig heavy chain V region 6.96	Tripartite motif-containing protein 60	
5	Proteasome subunit beta type-6	Annexin A7		Ig kappa chain V-V region MOPC 173		
6	Neuronal acetylcholine receptor subunit alpha-4	Calpain small subunit 1 (Fragment)		GTPase-activating protein and VPS9 domain-containing protein 1 (Fragment)		
7	Hemopexin	Pyrroline-5-carboxylate reductase 3		Loss of heterozygosity 12 chromosomal region 1 protein homolog 12		
8	Protein 1300017J02Rik	Protein Ahnak		Protein Shroom1		
9	Corticosteroid-binding globulin			Tubulin beta-1 chain		
10	Isoform 2 of Complement factor D			Tubulin alpha-4A chain		
11	Fructose-biphosphate aldolase (Fragment)			Tropomyosin alpha-4 chain		
12	Serine protease inhibitor A3N			Protein Epb4.1		
13	Protein Col6a3			Isoform 2 of Myosin-14		
14	Major urinary protein 8			Tropomyosin alpha-3 chain		
15	Histone H4			Platelet glycoprotein Ib beta chain		
16	Cytochrome P450 2D9			Trem-like transcript 1 protein		
17	Proteasome subunit beta type-4			Isoform M1 of Pyruvate kinase isozymes M1/M2		
18	Isoform 2 of Oncoprotein-induced transcript 3 protein			Profilin-1		
19	Formimidoyltransferase-cyclodeaminase			Myosin regulatory light polypeptide 9		
20	Proteasome subunit alpha type-4			Platelet-activating factor acetylhydrolase		
21	Major urinary protein 2			Serum deprivation-response protein		
22	Elongation factor 1-delta (Fragment)			Histone H1.1		
23	Latent-transforming growth factor beta-binding protein 1			Sarcoplasmic/endoplasmic reticulum calcium ATPase 3		
24	Ig alpha chain C region			Arachidonate 12-lipoxygenase, 12S-type		
25	Ferritin			L-lactate dehydrogenase A chain		
26	Proteasome subunit alpha type-3			ATP synthase subunit beta, mitochondrial		
27	Extracellular matrix protein 1			Isoform Short of Thymosin beta-4		
28	Erythrocyte band 7 integral membrane protein			Glycophorin-A		

Supplementary Table 11. Unique proteins on SMPN-1, -3, and -9 at 8 and 48 Hours (cont.).						
	8 Hours			48 Hours		
	SMPN-1	SMPN-3	SMPN-9	SMPN-1	SMPN-3	SMPN-9
**29**	Protein C7			Cofilin-1		
**30**	Cytochrome P450 2C29			Isoform MLC3 of myosin light chain 1/3, skeletal muscle isoform		
**31**	Mannose-binding protein A			Vinculin		
**32**	Protein Epb4.1			Malate dehydrogenase, mitochondrial		
**33**	Beta-2-glycoprotein 1			Aldehyde dehydrogenase, mitochondrial		
**34**	Ig kappa chain V-V region MOPC 173			Ig heavy chain V-III region J606		
**35**	Histone H3 (Fragment)			Ig gamma-2B chain C region		
**36**	Isoform 2 of Afamin			Isoform 2 of Spectrin alpha chain, non-erythrocytic		
**37**	60S ribosomal protein L35a			Isoform 3 of N-acetylmuramoyl-L-alanine amidase		
**38**	Protein C6			26S protease regulatory subunit 10B		
**39**	Ras-related protein Rap-1b			Bleomycin hydrolase		
**40**	Alpha-crystallin B chain			Ig alpha chain C region		
**41**	Isoform 2 of Spectrin alpha chain, non-erythrocytic					
**42**	Protein Ahnak2 (Fragment)					
**43**	Complement component 8, gamma subunit, isoform CRA_b					
**44**	Coagulation factor X					
**45**	Titin					
**46**	Ig lambda-2 chain V region					
**47**	Actin, alpha skeletal muscle					
**48**	Integrin beta-3					
**49**	Tyrosine-protein kinase BAZ1B					
**50**	ATP synthase subunit beta, mitochondrial					
**51**	Cofilin-1					
**52**	Protein Shroom1					

Supplementary Table 11. List of all unique proteins identified on SMPN-1, -3 and -9. Data is combined for four mice per group after 8 and 48 h *in vivo*. Proteins are ranked by abundance from highest to lowest. Summative data for each treatment group was generated by combining the proteins identified in each biological replicate to a full list of proteins identified on SMPNs collected within the molecular weight group and time point group. A complete dataset of proteins is given in Supplementary Data 1. Protein abundances are not provided here since protein abundances were taken as additive data from 4 different mice; however, the abundance of all individual identified proteins (N = 4 mice) are given in Supplementary Data 1.
