# Supplementary material for: Blood circulation of soft nanomaterials is governed by dynamic remodeling of protein opsonins at nano-biointerface
Source: Nat Commun. 2020 Jun 16;11:3048. doi: 10.1038/s41467-020-16772-x (PMC7298025; doi:10.1038/s41467-020-16772-x)
Supplement: Supplementary file 3 — Description of Additional Supplementary Files [file 41467_2020_16772_MOESM3_ESM.docx]

Description of Additional Supplementary Files

**Title:** Supplementary Data 1

**Description:** Details of proteins, intensity and raw data from proteomics studies
