# Supplementary material for: Blood circulation of soft nanomaterials is governed by dynamic remodeling of protein opsonins at nano-biointerface
Source: Nat Commun. 2020 Jun 16;11:3048. doi: 10.1038/s41467-020-16772-x (PMC7298025; doi:10.1038/s41467-020-16772-x)
Supplement: Supplementary file 5 — Reporting Summary [file 41467_2020_16772_MOESM5_ESM.pdf]

natureresearch

Corresponding author(s): Jayachandran N Kizhakkedathu

Last updated by author(s): May 12, 2020

Reporting Summary

Nature Research wishes to improve the reproducibility of the work that we publish. This form provides structure for consistency and transparency in reporting. For further information on Nature Research policies, see Authors & Referees and the Editorial Policy Checklist .

Statistics

For all statistical analyses, confirm that the following items are present in the figure legend, table legend, main text, or Methods section.

n/a	Confirmed	
☐	☒	The exact sample size (*n*) for each experimental group/condition, given as a discrete number and unit of measurement
☐	☒	A statement on whether measurements were taken from distinct samples or whether the same sample was measured repeatedly
☐	☒	The statistical test(s) used AND whether they are one- or two-sided<br>*Only common tests should be described solely by name; describe more complex techniques in the Methods section.*
☒	☐	A description of all covariates tested
☒	☐	A description of any assumptions or corrections, such as tests of normality and adjustment for multiple comparisons
☐	☒	A full description of the statistical parameters including central tendency (e.g. means) or other basic estimates (e.g. regression coefficient) AND variation (e.g. standard deviation) or associated estimates of uncertainty (e.g. confidence intervals)
☐	☒	For null hypothesis testing, the test statistic (e.g. *F*, *t*, *r*) with confidence intervals, effect sizes, degrees of freedom and *P* value noted<br>*Give P values as exact values whenever suitable.*
☒	☐	For Bayesian analysis, information on the choice of priors and Markov chain Monte Carlo settings
☒	☐	For hierarchical and complex designs, identification of the appropriate level for tests and full reporting of outcomes
☐	☒	Estimates of effect sizes (e.g. Cohen's *d*, Pearson's *r*), indicating how they were calculated

Our web collection on statistics for biologists contains articles on many of the points above.

Software and code

Policy information about availability of computer code

Data collection	CytExpert 2.3 [Beckman Coulter] was used for flow cytometry data collection, Slidebook 6.0 [3i] was used to collect confocal fluorescent microscopy images, and Bruker oTof Control 4.1 was used for mass spectroscopy data collection.
Data analysis	Prism Graphpad 7.0 was used for statistical data analysis and figure generation, Image J was used to quantify fluorescence from confocal microscopy images, MaxQuant 1.6.5.0 and Perseus 1.6.2.3 Data Visualization Softwares for proteomic data analysis were used. The database used was the Mus Musculus proteome downloadable from Uniprot.org; https://www.uniprot.org/proteomes/UP000000589.

For manuscripts utilizing custom algorithms or software that are central to the research but not yet described in published literature, software must be made available to editors/reviewers. We strongly encourage code deposition in a community repository (e.g. GitHub). See the Nature Research guidelines for submitting code & software for further information.

Data

Policy information about availability of data

All manuscripts must include a data availability statement. This statement should provide the following information, where applicable:

- Accession codes, unique identifiers, or web links for publicly available datasets
- A list of figures that have associated raw data
- A description of any restrictions on data availability

The mass spectrometry proteomic data have been deposited to the ProteomeXchange Consortium via the PRIDE partner MassIVE repository (UCSD, San Diego, CA, USA) with the data set identifier: PXD018958[https://doi.org/doi:10.25345/C5NX3V].

Field-specific reporting

Please select the one below that is the best fit for your research. If you are not sure, read the appropriate sections before making your selection.

☒ Life sciences ☐ Behavioural & social sciences ☐ Ecological, evolutionary & environmental sciences

For a reference copy of the document with all sections, see nature.com/documents/nr-reporting-summary-flat.pdf

Life sciences study design

All studies must disclose on these points even when the disclosure is negative.

Sample size	We chose four mice (N = 4) for all animal studies. This sample size is most commonly used for pharmacokinetic studies and proteomic studies and the corresponding citations were also listed in the manuscript [Leblanc et al. 2019; Bertrand et al. 2017]. For biocompatibility data using human specimens, n = 3 donors was selected, both as a standard in literature in the in vitro assessment of biocompatibility of materials, as well as to maintain consistency with previous publications (Abbina et al. 2019; Yu et al. 2014; Ahmed et al. 2012; Kainthan et al. 2006]
Data exclusions	No data were excluded in the analysis.
Replication	Standard deviations were reported for all in vivo and in vitro studies. For proteomic studies, average profile data from three technical replicates are shown for four different mice (biological replicates) are shown for each group. Reproducibility analysis (scatterplots and Pearson correlation between biological replicates for each treatment group at each time point) was conducted on Perseus Data Visualization Software by MaxQuant and is provided in the Supplementary Information. Unless otherwise mentioned, for all other studies, three experimental replicates were conducted, each with three technical replicates. For biocompatibility studies conducted in human blood, data was generated from three experimental replicates, using blood from 3 different donors, each with 3 technical replicates in order to minimize donor-to-donor variability.
Randomization	As no biases are anticipated with the selection of animals and treatments, sample randomization was not relevant towards are study and thus, no randomization protocols were used. For experiments involving human participants, we do not expect responses to biocompatibility to differ between blood collected from various sex, ethnic or age groups, and only blood from healthy donors, thus, sample randomization was not relevant and randomization procedures were not performed.
Blinding	As our study does not influence the participants, blinding is not relevant towards our study. Investigators were not blinded to the treatment groups as assignment of treatment group was necessary to correctly analyze pharmacokinetic, proteomic, and biocompatibility data.

Reporting for specific materials, systems and methods

We require information from authors about some types of materials, experimental systems and methods used in many studies. Here, indicate whether each material, system or method listed is relevant to your study. If you are not sure if a list item applies to your research, read the appropriate section before selecting a response.

Materials & experimental systems

n/a	Involved in the study	
☐	☒	Antibodies
☒	☐	Eukaryotic cell lines
☒	☐	Palaeontology
☐	☒	Animals and other organisms
☐	☒	Human research participants
☒	☐	Clinical data

Methods

n/a	Involved in the study	
☒	☐	ChIP-seq
☐	☒	Flow cytometry
☒	☐	MRI-based neuroimaging

Antibodies

Antibodies used	Mouse anti-human FITC-CD45 (Cat no. IM0782U, Lot No. 0000102, dilution: 1:40 , stock concentration: 50 ug/mL) was the only antibody used in this study.
Validation	Relevant validation information from the manufacturer can be found here: https://www.beckman.com/reagents/coulter-flow-cytometry/antibodies-and-kits/single-color-antibodies/cd45#f:@fformselected55615=[FITC].<br>Manufacturer states all the monoclonal antibodies that belong to the CD45 cluster react with and are able to recognize all CD45 isoforms. Demonstration of successful positive identification of leukocyte population in mice is demonstrated in the manuscript (Supplementary Fig. 5c) with identification purity >90%.

Animals and other organisms

Policy information about studies involving animals; ARRIVE guidelines recommended for reporting animal research

Laboratory animals	Mice (female and male, Balb/c, N = 4, 6 – 8 weeks) for proteomic studies and female Balb/c mice (N = 4, 6–8 weeks) for pharmacokinetic studies were used. Mice were sourced from Envigo. Mice were housed in cages in normal thermoneutral temperatures (between 24 - 26 degrees C) under stable 50% humidity conditions using light dark cycles of 12/12.
Wild animals	No wild animals were used in the study.
Field-collected samples	The study did not involve samples collected from the field.
Ethics oversight	The animal studies were conducted at the Experimental Therapeutics Laboratory at the British Columbia Cancer Research Centre, Vancouver, Canada. The protocol [A18-0276] was reviewed and approved by the Institutional Animal Care Committee (IACC) at the University of British Columbia."

Note that full information on the approval of the study protocol must also be provided in the manuscript.

Human research participants

Policy information about studies involving human research participants

Population characteristics	Blood from healthy and consenting male and female adult donors (ages 22 - 40) of varying ethnic backgrounds (Asian, European, South American) were used for this study.
Recruitment	Donors were recruited on a voluntary basis and donated at the Centre for Blood Research Blood Collection Suite. The participants were informed about the project though a "Participant Information and Consent Form", which donors were required to sign prior to donation.
Ethics oversight	Blood from healthy consented donors was either collected at Centre for Blood Research, University of British Columbia. The protocol was approved by clinical ethical committee of the University of British Columbia.

Note that full information on the approval of the study protocol must also be provided in the manuscript.

Flow Cytometry

Plots

Confirm that:

- [x] The axis labels state the marker and fluorochrome used (e.g. CD4-FITC).
- [x] The axis scales are clearly visible. Include numbers along axes only for bottom left plot of group (a 'group' is an analysis of identical markers).
- [x] All plots are contour plots with outliers or pseudocolor plots.
- [x] A numerical value for number of cells or percentage (with statistics) is provided.

Methodology

Sample preparation	Buffy coat fractions were isolated from the blood samples and washed 3 times with PBS. was used to identify the leukocyte population stained with FITC-labelled CD45 antibody (Immunotech, Cat No. A07782, Lot No. IM0782U, prepared at a 1:40 dilution) to assess uptake of labelled SMPNs by leukocytes in vivo.
Instrument	A CytoFLEX flow cytometer [Beckman Coulter] was used for the data collection.
Software	CytExpert Software 2.3 was used on Cytoflex flow cytometer [Beckman Coulter] for data collection and analysis.
Cell population abundance	10,000 cells collected per sample with over 90% cell population in abundance after sample processing (collection of buffy coat and washing of fractions 3X with PBS to remove platelets). To assure the purity of the cell population being assessed, naive leukocytes were used to set a gate for the data collection. Data were collectedonly for the cell population defined by the gate.
Gating strategy	Our goal was to assess update of nanoparticles in leukocytes, for the purpose of our study we were not (at this time) interested in addressing uptake by specific classes of leukocytes, thus multiple markers were not used. As a pan-leukocytic marker, otherwise known as leukocyte common antigen, discrimination between CD45 - and CD45 + cells was sufficient for these purposes to ensure limited contamination by other blood cells (RBC's and platelets).

- [x] Tick this box to confirm that a figure exemplifying the gating strategy is provided in the Supplementary Information.
